# Univariate-Guided Sparse Regression

**DOI:** 10.1162/99608f92.c79ff6db

**Published:** 2025-08-21

**Authors:** Sourav Chatterjee, Trevor Hastie, Robert Tibshirani

**Affiliations:** Statistics and Mathematics, Stanford University; Statistics and Biomedical Data Science, Stanford University; Statistics and Biomedical Data Science, Stanford University

**Keywords:** supervised learning, lasso, modeling

## Abstract

In this article, we introduce “uniLasso,” a novel statistical method for regression. This two-stage approach preserves the signs of the univariate coefficients and leverages their magnitude. Both of these properties are attractive for stability and interpretation of the model. Through comprehensive simulations and applications to real-world data sets, we demonstrate that uniLasso outperforms lasso in various settings, particularly in terms of sparsity and model interpretability. We prove asymptotic support recovery and mean-squared error consistency under a set of conditions different from the well-known irrepresentability conditions for the lasso. Extensions to generalized linear models (GLMs) and Cox regression are also discussed.

## Media Summary

We present a new statistical tool called “uniLasso” that improves how we build predictive models from data. This method is especially useful when trying to understand which factors are truly important in complex data sets—a task that arises in fields ranging from health research to economics.

The uniLasso algorithm works in two steps. First, it looks at how each variable relates to the outcome by itself, and it keeps track of whether the relationship is positive or negative. Then, it combines these relationships into a final model. This makes the results more stable and easier to interpret, which helps scientists and data analysts draw clearer conclusions from their data.

In tests on simulated and real-world data, uniLasso outperformed a popular method called the lasso, especially in choosing fewer, more meaningful variables without sacrificing accuracy. It also comes with solid mathematical guarantees under conditions that are easier to meet than those required by lasso. The method can be extended beyond basic regression to more complex settings, including models used for analyzing survival times and other common statistical frameworks.

In short, uniLasso offers a more interpretable and often more effective way to select key variables in data-driven research.

## 1. Introduction

High-dimensional regression and classification problems are ubiquitous across fields such as genomics, finance, and social sciences. In these settings, the lasso (least absolute shrinkage and selection operator) has emerged as a widely used methodology due to its ability to simultaneously perform variable selection and regularization, yielding sparse and interpretable models. Despite its widespread use, lasso has certain limitations, including sensitivity to correlated predictors and inclusion of spurious features in the final model. There have been many related proposals including MC+ (n.d.-a), the elastic net (n.d.-b), the adaptive lasso (n.d.-c), and SparseNet (n.d.-d).

To address these challenges, we propose “uniLasso,” a novel regression methodology. In this work, we aim to achieve two primary goals: predictive accuracy and (especially) interpretability. To achieve this, uniLasso integrates marginal (univariate) information into a coherent multivariate framework: our method preserves the signs of the univariate coefficients and leverages their magnitude. This approach not only enhances predictive accuracy but also provides insights into the relative importance of predictors.

Here is an example, taken from unpublished work with collaborators of the third author. (Since the work is unpublished, we do not give background details). The data set is from cancer proteomics, with 559 biomarkers measured on 81 patients, 20 healthy and 61 with cancer. We divided the data into 70% training and 30% test, and applied both the lasso and uniLasso to the training set. Both methods performed well, giving cross-validation error of about 5%, and four and three errors respectively out of 30 test samples. Table 1 shows the results of lasso and uniLasso applied to these data, using cross-validation to choose the model. In the top table there are two sign changes from univariate to multivariate: biomarker 12 goes from mildly positive to a negative indicator, while biomarker 11 goes in the opposite direction.

By design, uniLasso in the bottom table has no sign changes. Further, its univariate coefficients are much larger (in absolute value) than those of the lasso. This makes it a more credible and stable model.

The remainder of this article is organized as follows. Section 2 provides a detailed description of the uniLasso methodology. We examine the relationship between uniLasso and the adaptive lasso in Section 3, and in Section 4 we study the underlying reason for the strong sparsity produced by uniLasso. We compare uniLasso to lasso on a car price data set in Section 5: here we show the utility of unregularized uniLasso as an alternative to least squares. In Section 6 we look at the orthonormal design case, where we can derive an explicit expression for the uniLasso solution. Section 7 presents some theoretical results on support recovery and mean square error consistency. Section 8 presents simulation studies comparing uniLasso to lasso and other benchmark methods, while Section 9 examines *uniReg*, the unregularized (λ=0) case. Section 10 applies uniLasso to real-world data sets, and Section 11 explores further topics: in Section 11.1 we adapt uniLasso to multiclass classification problems; Section 11.2 explores the setting where additional data is available: not the complete data, but just the univariate scores; in Section 11.4 we study whether cross-validation works properly in the uniLasso setting; we discuss uniLasso for generalized linear models (GLMs) and the Cox survival model, as well as computation in Sections 11.5 and 11.6. Lastly, Section 12 concludes with a discussion of future directions.

## 2. Univariate-Guided lasso

### 2.1. Our Proposal

We assume the standard supervised learning setup: we have training features and target $X_{n\times p},\;y_{n\times 1}$. For now we assume that y is quantitative and we fit a linear model using squared error loss; later we discuss the binomial and other GLM families, as well as the Cox model.

Our procedure has three simple steps, which we motivate here. For interpretability and prediction accuracy, we preprocess the features in Step 1, multiplying them by a robust version of their univariate least-squares coefficient estimates. In Step 2 we fit a nonnegative lasso tuned by cross- validation, and Step 3 combines the components of Steps 1 and 2 to produce a final model. Together these ensure that:

- the signs of the final coefficients agree with the signs of the univariate coefficients (or they are zero);
- features with larger univariate coefficients will tend to have larger coefficients in the final model.

#### UniLasso Algorithm

1. For j=1,2,…,p compute the univariate intercepts and slopes $\left({\hat{\beta }}_{0j},{\hat{\beta }}_{j}\right)$ and their leave-one-out (LOO) counterparts $\left({\hat{\beta }}_{0j}^{-i},{\hat{\beta }}_{j}^{-i}\right),\;i=1,\;\ldots \;,n$.
2. Fit the lasso—with an intercept, no standardization, and nonnegativity constraints—to target y and the univariate LOO fits as features
$$\begin{array}{l} {minimize}_{\theta }\left\{\frac{1}{n}\sum_{i=1}^{n}{\left(y_{i}-\theta_{0}-\sum_{j=1}^{p}\left({\hat{\beta }}_{0j}^{-i}+{\hat{\beta }}_{j}^{-i}x_{ij}\right)\theta_{j}\right)}^{2}+\lambda \sum_{j=1}^{p}\theta_{j}\right\}\\ \;\text{subject}\;\text{to}\;\theta_{j}\geq 0\;\forall \;j>0. \end{array}$$
3. The final model can be written as $\hat{\eta }(x)={\hat{\gamma }}_{0}\sum_{j=1}^{p}{\hat{\gamma }}_{j}x_{j}$, with ${\hat{\gamma }}_{j}={\hat{\beta }}_{j}{\hat{\theta }}_{j}$, and ${\hat{\gamma }}_{0}={\hat{\theta }}_{0}+\sum_{\ell =1}^{p}{\hat{\beta }}_{0\ell }\;{\hat{\theta }}_{\ell }$.

This procedure is computationally convenient: in Step 1 we can use efficient LOO formulas, and in Step 2 we can apply any efficient $\ell_{1}$ solver. Here we use the R language package glmnet. Specifically, we use the function cv.glmnet to estimate the lasso path parameter, and have all of the functionality of glmnet at our disposal. We provide a function cv.uniLasso in the R package uniLasso that implements this approach.

Note that we *do not* standardize the features before applying the nonnegative lasso in Step 2; the univariate LOO fits are all on the scale of the response. From our knowledge of multiple linear regression, the first constraint—agreement between univariate and multivariate signs—may seem like an unreasonable restriction. However, our belief is that in high dimensions there are likely to be a multitude of different models that have about the same mean squared error (MSE) as the ‘optimal’ one chosen by the lasso. Hence it can make sense to choose one that is more interpretable and sparser than that of lasso.

These properties mean, for example, if the feature ‘age’ has a positive univariate coefficient—indicating increasing risk of the outcome variable (such as Alzheimer’s disease), it will have a positive (or zero) coefficient in the final lasso model. And if age is strongly significant on its own, it is more likely to be chosen in the multivariate model.

These conjectures are borne out by our numerical studies. In simulations with varying problems sizes and signal-to-noise ratio (SNR), and a number of real data sets (see Section 8), in almost every case uniLasso did no worse than the lasso in terms of out-of-sample MSE, and delivered a substantially sparser model. We note that (n.d.-e) studies sign-constrained least squares estimation for high-dimensional regression, which relates to our nonnegativity constraint in our second step.

##### Remark A.

The uniLasso procedure applies seamlessly to other GLMs as well the Cox model. Indeed, all of the models covered by the glmnet package are at our disposal. All we need are the separate fitted linear models and their LOO fit vectors in Step 1, and then glmnet can be directly applied. The only challenge is finding an (approximate) LOO formula for the GLM families. We give details of these computations in Section 11.6.

##### Remark B.

UniLasso can be thought of as a version of stacked regression, or *stacking* (n.d.-f); (n.d.-g). Stacking is a two-stage procedure for combining the predictions from a set of models (‘learners’), in order to get improved predictions. It works as follows. A set of base models is trained on the training data; these models can be of the same type (e.g., gradient boosting) or different types (e.g., linear regression, decision trees, etc.). Each model generates predictions, capturing specific aspects of the relationship between the predictors and the target variable. A meta-model is then trained to combine the predictions of the base models into a final prediction. This meta-model learns how to weigh and integrate the LOO outputs of the base models to minimize the overall prediction error. UniLasso is a special case of stacking where the individual learners are simple univariate regressions.

##### Remark C.

Why do we use the LOO univariate estimates ${\hat{\eta }}_{j}^{-i}={\hat{\beta }}_{0j}^{-i}+{\hat{\beta }}_{j}^{-i}x_{ij}$ as features in Step 2, instead of the univariate estimates ${\hat{\eta }}_{j}^{i}={\hat{\beta }}_{0j}+{\hat{\beta }}_{j}x_{ij}$?^1^ In traditional stacking this is essential because the individual learners can be of very different complexity. In uniLasso, one would think that the learners (univariate regression) all have the same complexity so that the LOO versions are not needed. Indeed, the theory in Section 7 is based on the LOO estimates but holds equally well for the usual (non-LOO) estimates. However, in practice we have found that the LOO estimates lead to greater sparsity and better performance, and hence we use them. With the use of the usual (non-LOO) univariate estimates in uniLasso, the resulting estimator is closely related to the adaptive lasso and we explore this connection in Section 3.

##### Remark D.

The nonnegative garotte (n.d.-h) is another closely related method. It minimizes $\sum_{i}{\left(y_{i}-\sum_{j}c_{j}{\hat{\beta }}_{j}x_{ij}\right)}^{2}$ subject to $c_{j}\geq 0$ for all j, and $\sum c_{j}\leq s$ where ${\hat{\beta }}_{j}$ are the usual (multivariable) LS estimates. This is different from our proposal in an important way: our use of univariate LOO estimates in the first step.The univariate coefficients lead to materially different solutions and also allow application to the p>n scenario.

### 2.2. UniLasso With No Regularization

We get an interesting special case of uniLasso if we set λ=0, so that there is no $\ell_{1}$ regularization in Step 2. That is, we solve the following:

(2.1) $${minimize}_{\theta }\left\{\frac{1}{n}\sum_{i=1}^{n}{\left(y_{i}-\theta_{0}-\sum_{j=1}^{p}\theta_{j}\left({\hat{\beta }}_{0j}^{-i}+{\hat{\beta }}_{j}^{-i}x_{ij}\right)\right)}^{2}\right\}\;\text{subject}\;\text{to}\;\theta_{j}\geq 0\;\forall j>0$$

We call this ‘uniReg’ for univariate-guided regression and it represents an interesting alternative to the usual least squares estimates. For example, the nonnegative constraint can still provide sparsity even though there is no $\ell_{1}$ penalty. If p>n there can be multiple solutions; in this case we look for the sparsest solution by computing the limiting uniLasso solution as λ↓0. The standard error and distribution of the estimated coefficients can be estimated via the bootstrap. We study uniReg in detail in Section 9.

### 2.3. Two Examples—One Favorable, One Not

#### 2.3.1. Homecourt

We generated data with 100 observations, 30 standard normal features with AR(1) correlation 0.8, 20% sparsity, and nonnegative coefficients in two stages, as follows:

(2.2) $${y_{i}}^{\prime }\leftarrow \sum_{j}x_{ij}\beta_{j}+\sigma^{\prime }{z_{i}}^{\prime }$$

From these data we compute the p=30 univariate least squares coefficients ${\hat{\beta }}_{j}^{\mathrm{uni}}$ separately for each j, and then generate y as

(2.3) $$y_{i}\leftarrow \sum_{j}x_{ij}{\hat{\beta }}_{j}^{uni}\beta_{j}+\sigma z_{i}.$$

In the above, ${z_{i}}^{\prime },\;z_{i}$ are standard normal variates, and the variance terms $\sigma^{\prime }$ and σ were chosen so that at both stages the SNR was 1. The idea here is that (2.3) roughly mimics the model fit by uniLasso in its second stage.

**Figure 1.**
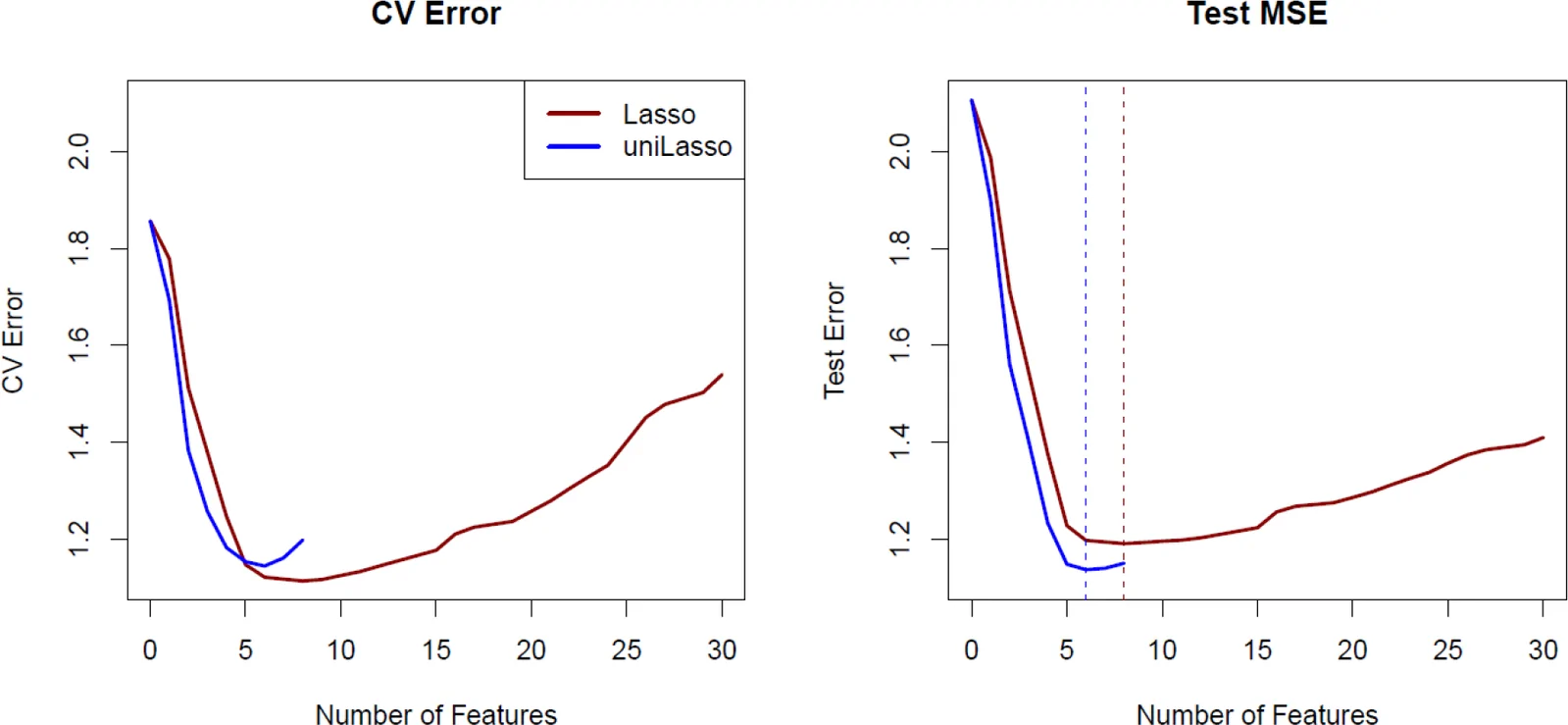
Results for homecourt example: CV and test set prediction error. The dashed vertical lines for each method correspond to the models chosen by CV. CV = cross-validation.

Figure 1 shows the cross-validation (CV) and test error curves for the lasso and uniLasso. We see that uniLasso has test error a little below that of lasso, with a smaller active set. Table 2 shows the result of 100 simulations from this setup. We see that the same pattern emerges, and uniLasso exhibits a slightly lower true positive rate and a much lower false positive rate than the lasso.

#### 2.3.2. Counter-Example

Here we take $n=100,\;p=20,\;x_{1}∼N(0,1)$, $x_{2}=x_{1}+N(0,1)$, β=(1,-.5,0,0,…0), and error SD=0.5. The remaining 18 features are standard normal. This is an ‘Achilles heel’ for uniLasso, as the (negative) sign of the population coefficient for $x_{2}$ differs from its (positive) univariate sign. As a result, the test MSE for uniLasso is about twice that of lasso (detailed results are given in Section 8). Clearly uniLasso fails badly here, but its high CV error will alert the user to this. Motivated by this kind of example, we discuss a postprocessing (‘polish’) for uniLasso in Section 11.3 that remedies this problem.

## 3. Relationship of uniLasso to the Adaptive lasso

The adaptive lasso (n.d.-i) is defined by

(3.1) $${\hat{\gamma }}^{*}=argmin\frac{1}{n}\sum_{i=1}^{n}{\left(y_{i}-\gamma_{0}-\sum_{j=1}^{p}x_{ij}\gamma_{j}\right)}^{2}+\lambda \sum_{j=1}^{p}w_{j}\left|\gamma_{j}\right|$$

where $w_{j}=1/{\left|{\hat{\gamma }}_{j}\right|}^{v}$. Here the vector $\hat{\gamma }$ is any root-n consistent estimate of γ, for example, the least squares estimates.

The original paper assumed n>p; with p≥n, one cannot use the least-squares estimates to define w. One possibility is to use an initial estimate (from say ridge or lasso), solve the adaptive lasso, and iterate. Another option is to use the univariate least-squares estimates; (n.d.-j) consider a combination of these to achieve some theoretical guarantees.

Let ${\left\{{\hat{\beta }}_{j}\right\}}_{1}^{p}$ be the univariate least squares coefficients. Consider the adaptive lasso with weights $w_{j}=1/\left|{\hat{\beta }}_{j}\right|,\;j=1,\;\ldots \;,p$. Then it is easy to show that this procedure is equivalent to uniLasso with ${\hat{\eta }}_{j}^{i}={\hat{\beta }}_{0j}+{\hat{\beta }}_{j}x_{ij}$ replacing their LOO versions ${\hat{\eta }}_{j}^{-i}={\hat{\beta }}_{0j}^{-i}+{\hat{\beta }}_{j}^{-i}x_{ij}$ in Step 2, and removing the nonnegativity constraint.

This procedure does not share some of the properties of uniLasso. In particular, the final coefficients may not have the same signs as the univariate coefficients. Importantly, in our simulations of Section 8, it tends to yield less sparse models and sometimes much higher MSE.

Alternatively, one could include the nonnegativity constraints in this version of the adaptive lasso. The following result shows that we obtain a procedure equivalent to uniLasso, except for the use of LOO estimates in Step 1.

### Proposition 1.

Let $\left({\hat{\beta }}_{0j},{\hat{\beta }}_{j}\right)$ be the univariate least squares coefficients for variable j in a p variable linear model with data ${\left\{\left(x_{i},y_{i}\right)\right\}}_{1}^{n}$. Let $\eta_{j}^{i}={\hat{\beta }}_{0j}+{\hat{\beta }}_{j}x_{ij}$ be the univariate linear fit for variable j and observation i.

Then the following two problems are equivalent:

(3.2) $$\underset{\theta }{min}\sum_{i=1}^{n}{\left(y_{i}-\theta_{0}-\sum_{j=1}^{p}\eta_{j}^{i}\theta_{j}\right)}^{2}+\lambda \sum_{j=1}^{p}\left|\theta_{j}\right|\;\text{s.t.}\;\theta_{j}\geq 0\;\forall j$$

(3.3) $$\underset{\gamma }{min}\sum_{i=1}^{n}{\left(y_{i}-\gamma_{0}-\sum_{j=1}^{p}x_{ij}\gamma_{j}\right)}^{2}+\lambda \sum_{j=1}^{p}\frac{\left|\gamma_{j}\right|}{\left|{\hat{\beta }}_{j}\right|}\;\text{s.t.}\;sign\left(\gamma_{j}\right)=sign\left({\hat{\beta }}_{j}\right)\;\forall j$$

The exact equivalence is obtained with ${\hat{\gamma }}_{j}={\hat{\theta }}_{j}{\hat{\beta }}_{j}$, and ${\hat{\gamma }}_{0}={\hat{\theta }}_{0}+\sum_{\ell =1}^{p}{\hat{\theta }}_{\ell }\;{\hat{\beta }}_{0\ell }$.

The proof is in Appendix A. However, our experiments with this version of adaptive lasso produced models that were not nearly as sparse as the ones using LOO. In fact, in Table 3 we see that enforcing sign constraints led to less sparse models than not enforcing sign constraints!

Finally we note that (n.d.-k) propose an iterated version of the adaptive lasso, in which the current solutions are used to define weights for the next iteration. They show that the method can enhance sparsity, especially for signal recovery.

Next we examine the relative performance of uniLasso when we modify the three main aspects of its design:

1. the use of LOO features ${\hat{\eta }}_{j}^{-i}$ versus non-LOO features ${\hat{\eta }}_{j}^{i}$,
2. the sign constraints, and
3. the scaling in Step 2 by the magnitude of the univariate coefficients, rather than just by their signs.

Note that the adaptive lasso is equivalent to uniLasso using non-LOO estimates and no sign constraints.

Table 3 shows the results of 50 simulation runs with n=300,p=1,000, and SNR = 1, our ‘medium-SNR’ setting detailed in Section 8.

For noMag, we first flip the signs of the columns of the feature matrix X to agree with the univariate coefficients. and call it $X_{s}$. We then compute the univariate fit matrix (columnwise), and its LOO counterpart; wherever they differ elementwise in sign, we flip the sign of the corresponding element in $X_{s}$ yielding $X_{\mathrm{loo}}$. The top table uses LOO univariate coefficients (as in uniLasso) while the bottom table uses the usual (non-LOO) versions.

We see that there is not much difference in test error, but uniLasso produces the sparsest models by a large margin. We also see the curious result that with no LOO, the uniLasso *without* sign constraints selects sparser models than *with* sign constraints.

## 4. Why Does the uniLasso Produce Such Sparse Solutions?

As we saw in the previous section, the combination of LOO univariate estimates in Step 1 and nonnegativity constraints in Step 2 seem to be the key to the strong sparsity delivered by uniLasso. What is the explanation for this? Our analysis focuses on the special case of the uniReg (λ=0) case.^2^

The correlation between the responses $y_{i}$ and the univariate fitted values ${\hat{\beta }}_{0j}+{\hat{\beta }}_{j}x_{ij}$ is always positive, and is the absolute value of the correlation between y and the *j*th feature. When this correlation is large, then the correlation between $y_{i}$ and the LOO fitted values ${\hat{\beta }}_{0j}^{-i}+{\hat{\beta }}_{j}^{-i}x_{ij}$ will also tend to be positive. But if the first correlation is positive and small, then the second correlation is often negative. This could be explained by the fact that leaving an observation out ‘pushes’ the fitted regression line away from that point, and if there was not much correlation to start with, that will be enough to tip the correlation of the LOO feature to be negative.^3^

**Figure 2.**
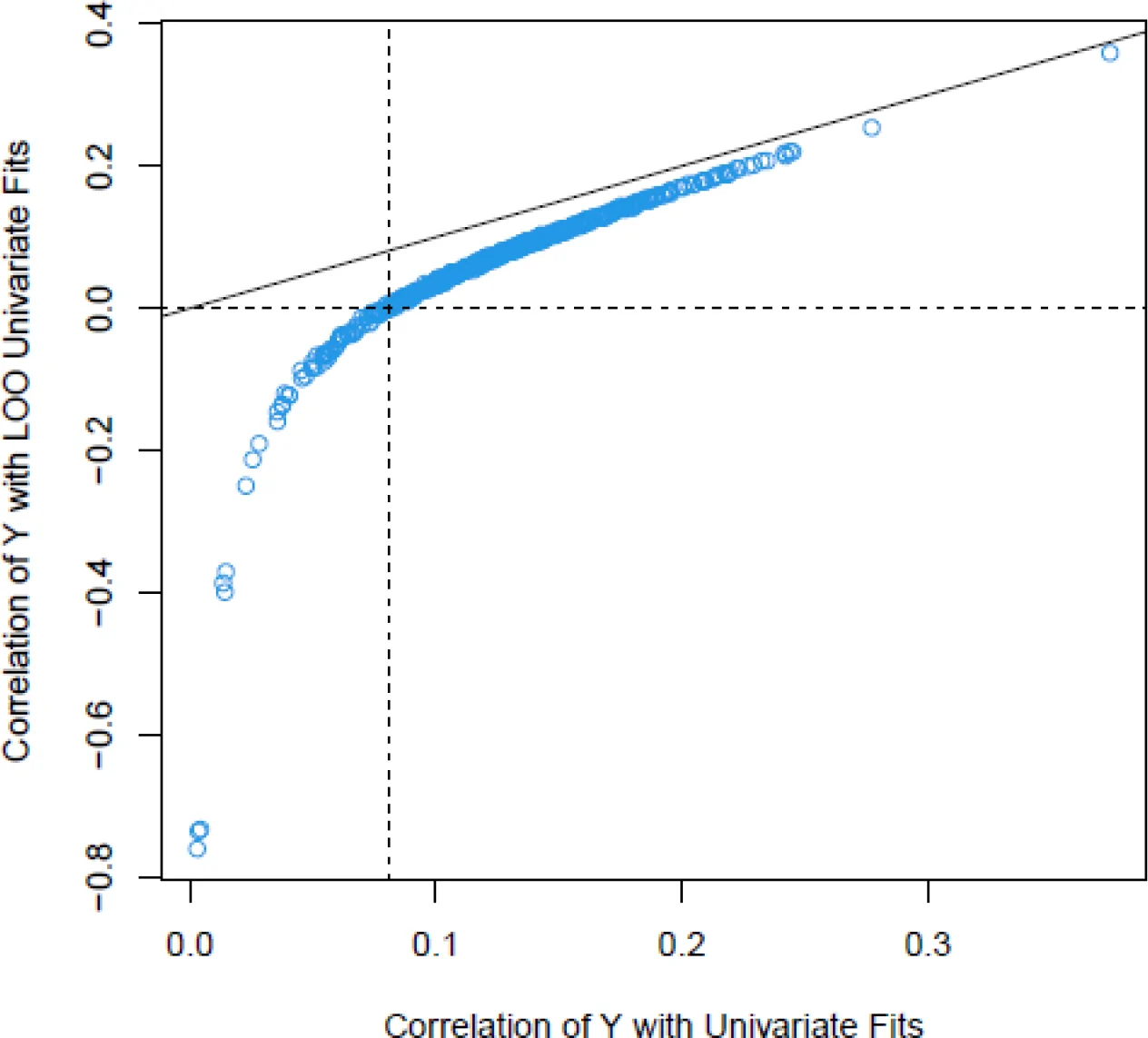
Medium-SNR setting: correlation between the response and the univariate LOO features, versus the correlation between the response and the usual univariate (non-LOO) features. The solid line represents equality.

Figure 2 shows an example from our ‘medium-SNR’ setting with n=300,p=1,000. The correlations between y and each feature needs to be larger than about 0.08 in order for the correlation between y and each LOO feature to also be positive.

In Appendix B we show that if the absolute correlation between y and a feature is smaller than approximately $\sqrt{2/n}$, then the correlation with the LOO fit will be negative. Since we have n=300 in this example, this evaluates to 0.082. Asymptotically, this means that the two-sided *p*-value for testing p=0 has to be greater than about 0.16.

In the second step of the uniLasso algorithm in Section 2.1, we fit a multivariate lasso model using all the LOO features, but with a positivity constraint on their coefficients. So even though marginally some LOO features are negatively correlated with the response, this does not guarantee that they will be omitted; however, in practice they do tend to be omitted.

## 5. Application to the Car Prices Data

This data set consists of 25 features for predicting prices for 205 cars, taken from https://www.kaggle.com/datasets/hellbuoy/car-price-prediction.

Make of car (22 levels) was first fit to the data and we modeled the residual on the remaining 26 predictors, 11 of which were categorical (and one-hot encoded).

Table 4 shows the univariate least squares coefficients on the left, and the lasso and uniLasso coefficients in the middle columns. The right column shows the ‘uniLasso polish’ estimates described in Section 11.3. They result from a postprocessing of uniLasso in which the lasso is applied to the uniLasso residuals. Only features having a nonzero coefficient in at least one of the three rightmost columns are shown. We see that uniLasso produces a much sparser model than the lasso.

**Figure 3.**
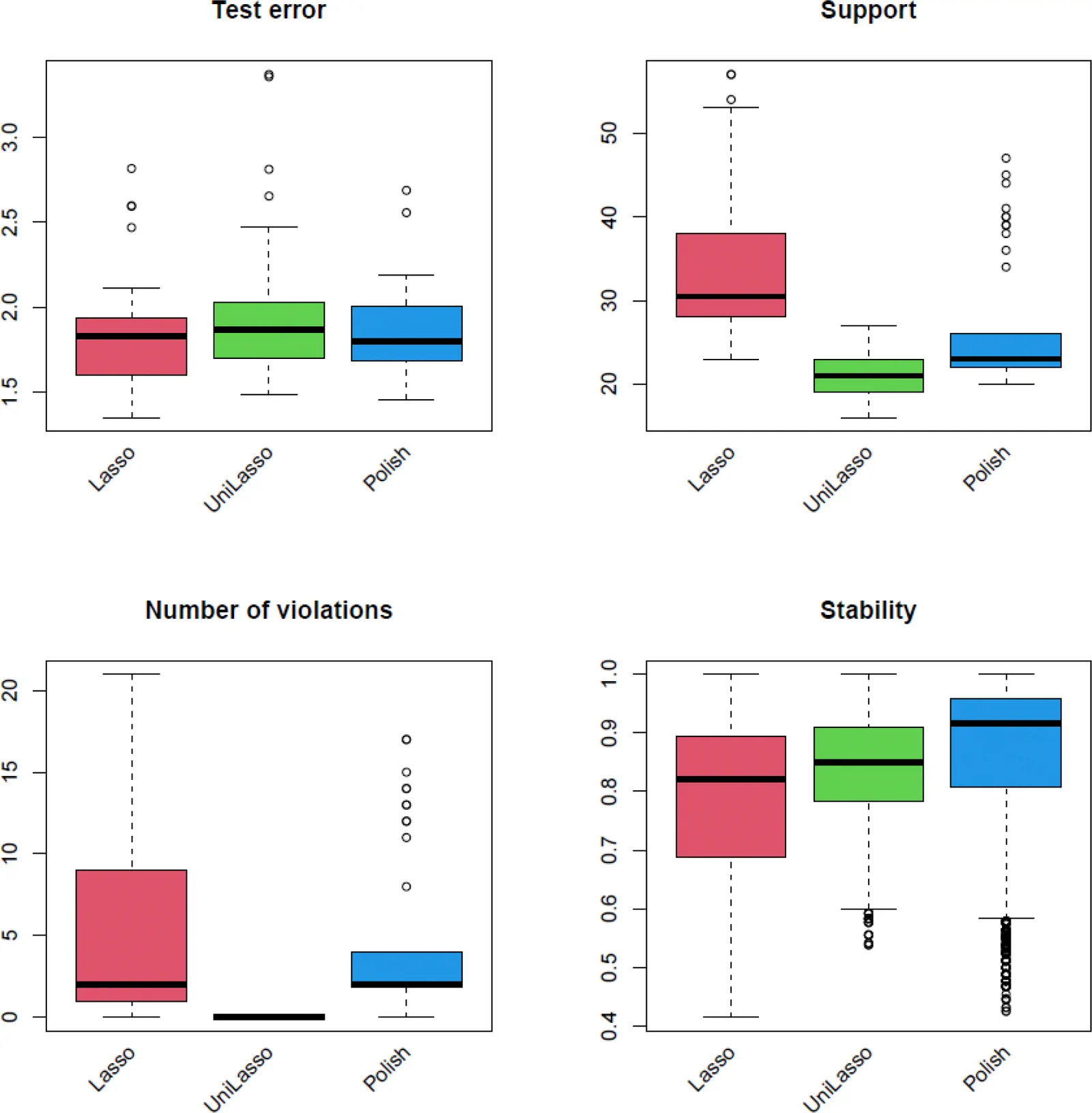
Results for car prices data over 50 train-test splits. Shown are test set error, size of the chosen model (support), number of sign change violations relative to the univariate signs, and the stability—the average proportion of common features over all model pairs for each method.

For Figure 3 we took 50 random 2/3–1/3 train-test splits, and computed four summaries of model performance. We see that the test errors of all methods are similar, while the support size and number of sign violations are quite different, as expected. The stability figure is most interesting: for each of the 502 pairs of models, we defined the ‘stability’ as ratio of the number of chosen features in common, divided by the number of unique features in the union of the pair. We see an advantage for uniLasso over lasso. The uniLasso ‘polish,’ described in Section 11.3 gives a further improvement instability, while increasing the support and number of sign violations.

## 6. Analysis of uniLasso With Orthogonal Features

**Figure 4.**
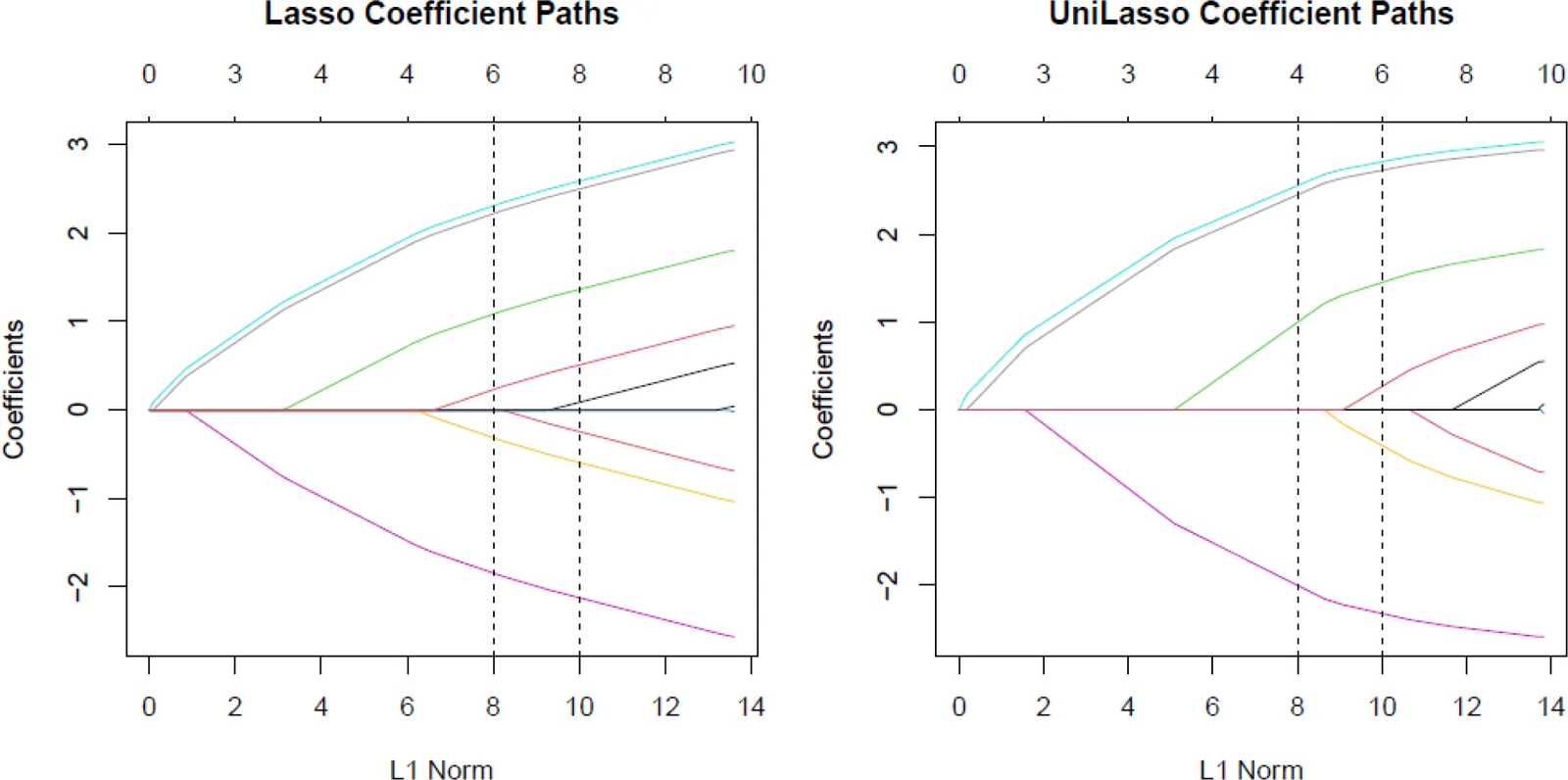
Coefficient paths for lasso and uniLasso, in a simulated example with 10 orthonormal features. The end of the path represents the least squares fit, and in this case also the univariate coefficients. The paths for features with large absolute univariate coefficients look very similar in both plots. Those with small univariate features have a delayed entry in the right plot.

In this section we derive an explicit formula for the uniLasso coefficients in the special case of orthonormal features. Figure 4 shows the lasso and uniLasso paths for a simulated example with an orthonormal feature matrix. They look somewhat different, with the uniLasso path being sparser at any stage along the path (as measured by the $\ell_{1}$ norm of the coefficients).

It is well-known that in this setting, the lasso coefficients are simply soft-thresholded versions of the univariate least squares estimates. This is (approximately) true of uniLasso, but with a different thresholding function, as we now show.

Suppose X is orthonormal so that each column $x_{j}$ satisfies ${\left\|\;x_{j}\;\right\|}_{2}=1$ and $x_{i}^{T}x_{j}=0$ for (i≠j). Assume also that $\bar{y}=0$ and ${\bar{x}}_{j}=0$ for each j. Note that the least squares coefficients are ${\hat{\beta }}_{0j}=0$ and ${\hat{\beta }}_{j}=x_{j}^{T}y$.

Here we use the actual fits ${\hat{\eta }}_{j}^{i}$ rather than the LOO fits ${\hat{\eta }}_{j}^{-i}$ in the second stage—an approximation that simplifies the derivation, and often gives very similar solutions to uniLasso. In this case we have ${\hat{\eta }}_{j}^{i}={\hat{\beta }}_{j}x_{ij}$, since ${\hat{\beta }}_{0j}=0$. We can ignore $\theta_{0}$, which will be zero since $\bar{y}=0$ and all the ${\hat{\eta }}_{j}={\hat{\beta }}_{j}x_{j}$ have means zero.

The coefficients ${\hat{\theta }}_{j}$ are determined by solving:

$$\underset{\theta_{j}}{min}\left\{\frac{1}{2}{\left\|\;y-\sum_{\ell =1}^{p}\theta_{\ell }\;{\hat{\beta }}_{\ell }x_{\ell }\;\right\|}_{2}^{2}+\lambda \;{\left\|\;\theta \;\right\|}_{1}\right\}$$

We have the derivative with respect to $\theta_{j}$:

$$-{\hat{\beta }}_{j}x_{j}^{⊤}\left(y-\sum_{\ell =1}^{p}\theta_{\ell }\;{\hat{\beta }}_{\ell }x_{\ell }\right)+\lambda \cdot sign\left(\theta_{j}\right)=0$$

Since the $x_{j}$ are orthogonal and unit norm, and using $x_{j}^{⊤}y={\hat{\beta }}_{j}$, we get ${{\hat{\beta }}_{j}}^{2}\theta_{j}={\hat{\beta }}_{j}^{2}-\lambda \cdot sign\left(\theta_{j}\right)$, and hence

$$\theta_{j}={\left(1-\frac{\lambda }{{\hat{\beta }}_{j}^{2}}\right)}_{+}.$$

The final coefficients for $x_{j}$ are

(6.1) $${\hat{\gamma }}_{j}={\hat{\theta }}_{j}\cdot {\hat{\beta }}_{j}={\hat{\beta }}_{j}{\left(1-\frac{\lambda }{{\hat{\beta }}_{j}^{2}}\right)}_{+}=sign\left({\hat{\beta }}_{j}\right){\left(\left|{\hat{\beta }}_{j}\right|-\frac{\lambda }{\left|{\hat{\beta }}_{j}\right|}\right)}_{+}$$

The last expression can be compared with the similar expression for the lasso in this situation:

(6.2) $$sign\left({\hat{\beta }}_{j}\right){\left(\left|{\hat{\beta }}_{j}\right|-\lambda \right)}_{+}$$

**Figure 5.**
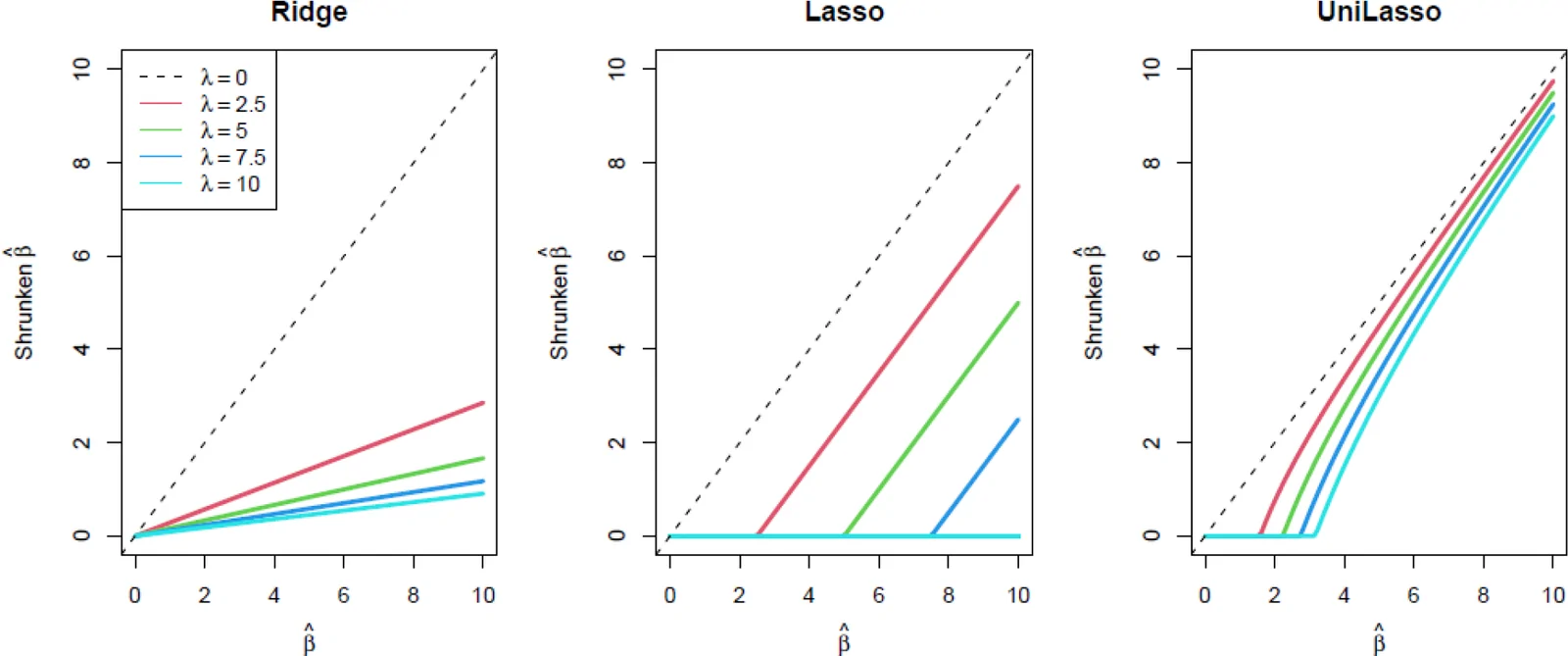
Shrinkage functions for ridge regression, lasso and uniLasso, in a simulated example with 10 orthonormal features.

Figure 5 shows the shrinkage functions for ridge regression, lasso, and uniLasso. Ridge uses proportional shrinkage, while lasso translates all coefficient to zero by the same amount. UniLasso is similar to lasso, except that the larger coefficients are shrunk less than the smaller ones.

The SparseNet procedure (n.d.-l) uses a thresholding function that has a roughly similar shape to that of uniLasso, but its objective is not convex. This shrinkage pattern is somewhere between lasso and $\ell_{0}$ or best subset selection. UniLasso achieves this without losing convexity, a significant computational advantage.

## 7. Theoretical Analysis

In this section we study the support recovery and mean-squared error properties of uniLasso. Let $X_{1},\;\ldots \;,X_{p}$ be square-integrable random variables with nonzero variance, defined on the same probability space, and let

$$Y=\gamma_{0}+\sum_{j\in S}\gamma_{j}X_{j}+\epsilon ,$$

where S is a subset of {1,…,p}, ϵ is a mean zero random variable that is independent of the $X_{j}$'s, and the ${\left(\gamma_{j}\right)}_{j\in S}$ are nonzero coefficients. We will refer to S as the *support*. Assume that Y also has nonzero variance. Our data consists of independent and identically distributed random vectors $\left(Y_{i},X_{i,1},\;\ldots \;,X_{i,p}\right),\;i=1,\;\ldots ,n$ (where n≥2), each having the same distribution as $\left(Y,X_{1},\;\ldots \;,X_{p}\right)$.

The uniLasso algorithm with penalty parameter λ>0 goes as follows. For each 1≤i≤n and 1≤j≤p, let

$${\hat{\beta }}_{j}^{-i}:=\frac{\frac{1}{n\;-\;1}\sum_{k\neq i}Y_{k}X_{k,j}\;-\;\left(\frac{1}{n\;-\;1}\sum_{k\neq i}Y_{k}\right)\left(\frac{1}{n\;-\;1}\sum_{k\neq i}X_{k,j}\right)}{\frac{1}{n\;-\;1}\sum_{k\neq i}X_{k,j}^{2}\;-\;{\left(\frac{1}{n\;-\;1}\sum_{k\neq i}X_{k,j}\right)}^{2}}$$

be the regression coefficient from the univariate regression of Y on $X_{j}$ omitting observation i, and let

$${\hat{\alpha }}_{j}^{-i}:=\frac{1}{n\;-\;1}\sum_{k\neq i}Y_{k}-\frac{{\hat{\beta }}_{j}^{-i}}{n\;-\;1}\sum_{k\neq i}X_{k,j}$$

be the intercept term. Note that ${\hat{\beta }}_{j}^{-i}$ is a consistent estimate of

$$\beta_{j}:=\frac{Cov\left(Y,\;X_{j}\right)}{Var\left(X_{j}\right)}$$

and ${\hat{\alpha }}_{j}^{-i}$ is a consistent estimate of

$$\alpha_{j}:=E(Y)-\beta_{j}E\left(X_{j}\right).$$

Then, let

$${\hat{Y}}_{i,j}:={\hat{\alpha }}_{j}^{-i}+{\hat{\beta }}_{j}^{-i}X_{i,j}$$

be the predicted value of $Y_{i}$ from this univariate regression. Next, obtain the estimates ${\hat{\theta }}_{j},\;j=0,\;\ldots ,p$, by minimizing

$$L\left(\theta_{1},\;\ldots \;,\theta_{p}\right)=\frac{1}{n}\sum_{i=1}^{n}{\left(Y_{i}-\theta_{0}-\theta_{1}{\hat{Y}}_{i,1}-\cdots -\theta_{p}{\hat{Y}}_{i,p}\right)}^{2}+\lambda \sum_{j=1}^{p}\theta_{j}$$

subject to the constraint that $\theta_{j}\geq 0$ for each 1≤j≤p. Finally, define

$${\hat{\gamma }}_{j}:={\hat{\theta }}_{j}{\hat{\beta }}_{j},$$

to be the uniLasso estimate of $\gamma_{j}$ for 1≤j≤p, where

$${\hat{\beta }}_{j}:=\frac{\frac{1}{n}\sum_{k=1}^{n}Y_{k}X_{k,j}\;-\;\left(\frac{1}{n}\sum_{k=1}^{n}Y_{k}\right)\left(\frac{1}{n}\sum_{k=1}^{n}X_{k,j}\right)}{\frac{1}{n}\sum_{k=1}^{n}X_{k,j}^{2}\;-\;{\left(\frac{1}{n}\sum_{k=1}^{n}X_{k,j}\right)}^{2}}$$

is the regression coefficient from the univariate regression of Y on $X_{j}$, and let

$${\hat{\gamma }}_{0}:={\hat{\theta }}_{0}+\sum_{j=1}^{p}{\hat{\theta }}_{j}{\hat{\alpha }}_{j},$$

where

$${\hat{\alpha }}_{j}:=\frac{1}{n}\sum_{k=1}^{n}Y_{k}-\frac{{\hat{\beta }}_{j}}{n}\sum_{k=1}^{n}X_{k,j}$$

is the intercept term from the univariate regression of Y on $X_{j}$.

The following theorem shows, roughly speaking, that if (1) $sign\left(\gamma_{j}\right)=sign\left(\beta_{j}\right)$ for each j∈S, (2) the penalty parameter λ is bigger than $\left|\beta_{j}\right|$ for all j∉S∪{0}, and (3) both logp and logn are small compared to $n\lambda^{2}$, then with high probability, ${\hat{\gamma }}_{j}=0$ for all j∉S∪{0} and ${\hat{\gamma }}_{j}=\gamma_{j}+O(\lambda )$ for all j∈S∪{0}.

### Theorem 1.

Suppose that:

1. $\gamma_{j}\beta_{j}>0$ *for each* j∈S.
2. The covariance matrix of ${\left(X_{j}\right)}_{j\in S}$ is nonsingular with minimum eigenvalue η.
3. *There is a positive constant* $C_{0}$ *such that* $Var(Y)\geq C_{0}$ *and* $Var\left(X_{j}\right)\geq C_{0}$ *for each* j∈S.
4. *There are positive constants* $C_{1}$ *and* $C_{2}$ *such that for each* t≥0 *and* 1≤j≤p,P(|Y|≥t), P(|ϵ|≥t) *and* $P\left(\left|X_{j}\right|\geq t\right)$ *are bounded above by* $C_{1}e^{-C_{2}t^{2}}$.

*Let* $M_{1}:={max}_{j\in S\cup \{0\}}\left|\gamma_{j}\right|,\;M_{2}:={min}_{j\in S}\left|\beta_{j}\right|$ *and* $M_{3}:={max}_{j\in S}\left|\beta_{j}\right|$. *Then there are positive constants* $K_{1},\;K_{2},\;K_{3},\;K_{4},\;K_{5}$ *depending only on* $C_{0},\;C_{1},\;C_{2},\;\eta ,\;M_{1},\;M_{2},\;M_{3}$ *and* |S| *such that if*

$$K_{1}\underset{j\notin S\cup \{0\}}{max}\left|\beta_{j}\right|\leq \lambda \leq K_{2},$$

then

$$\begin{array}{l} P\left({\hat{\gamma }}_{j}\right.\left.=0\;\text{for}\;\text{all}\;j\notin S\cup \{0\}\;\text{and}\;\left|{\hat{\gamma }}_{j}-\gamma_{j}\right|\leq K_{3}\lambda \;\text{for}\;\text{all}\;j\in S\cup \{0\}\right)\\ \;\geq 1-K_{4}pne^{-K_{5}n\lambda^{2}}. \end{array}$$

The above theorem is roughly comparable to the available results for the lasso. A close comparison would be, for instance, (n.d.-m). Like Theorem 1, this theorem also assumes a lower bound on the covariance matrix of the covariates in the support, and the maximum difference between ${\hat{\gamma }}_{j}$ and $\gamma_{j}$ for j∈S is of order λ. However, there is one key difference. The results about lasso, including the one cited above, require a condition known as *mutual incoherence or irrepresentability*. Roughly speaking, it means that if we regress $X_{k}$ for some k∉S on ${\left(X_{j}\right)}_{j\in S}$, the regression coefficients should be small. Notably, our Theorem 1 requires no such relation to hold between the covariates inside and outside the support. All we need is that the univariate regression coefficients of Y on covariates outside the support are small.

Having said that, we make clear that assumption (1) above is a crucial one: namely that $\gamma_{j}$ and $\beta_{j}$ have the same sign for each j∈S. The next result gives a natural sufficient condition under which this holds.

### Theorem 2.

*Let* $\delta_{j,k}$ *denote the population value of the univariate regression of* $X_{j}$ *on* $X_{k}$. *Suppose that for every pair of distinct indices* $j,\;k\in S,\;\delta_{j,k}\geq 0$ *if* $\gamma_{j}$ *and* $\gamma_{k}$ *have the same sign, and* $\delta_{j,k}\leq 0$ *if* $\gamma_{j}$ *and* $\gamma_{k}$ *have opposite signs. Then* $\beta_{j}$ *is nonzero and has the same sign as* $\gamma_{j}$ *for each* j∈S. *Moreover*, $\left|\beta_{j}\right|\geq \left|\gamma_{j}\right|$ *for each* j∈S.

The last result, partly due to Ryan Tibshirani, gives a necessary and sufficient condition for equality of signs.

### Theorem 3.

*Suppose that* $Y=\sum_{j\in S}\gamma_{j}X_{j}+\epsilon$, *where all the* $\gamma_{j}$*'s are nonzero, and* ϵ *has zero mean, finite variance, and is independent of* ${\left(X_{j}\right)}_{j\in S}$. *Let* $\beta_{j}$ *be the coefficient of* $X_{j}$ *in the univariate (population) regression of* Y *on* $X_{j}$. *Let* $\delta_{kj}$ *be the coefficient of* $X_{j}$ *in the univariate (population) regression of* $X_{k}$ *on* $X_{j}$. *For each* j, *let* $A_{j}$ *be the set of* k∈S *for which* $sign\left(\delta_{kj}\right)=sign\left(\gamma_{k}\gamma_{j}\right)$ *or* $\delta_{kj}=0$. *Then* $\beta_{j}\gamma_{j}\geq 0$ *if and only if*

$$\sum_{k\notin A_{j}}\left|\gamma_{k}\delta_{kj}\right|\leq \sum_{k\in A_{j}}\left|\gamma_{k}\delta_{kj}\right|.$$

In particular, if $A_{j}=S$, then this holds.

The proofs of these results are in Appendix C.

## 8. Some Simulation Results

**Figure 6.**
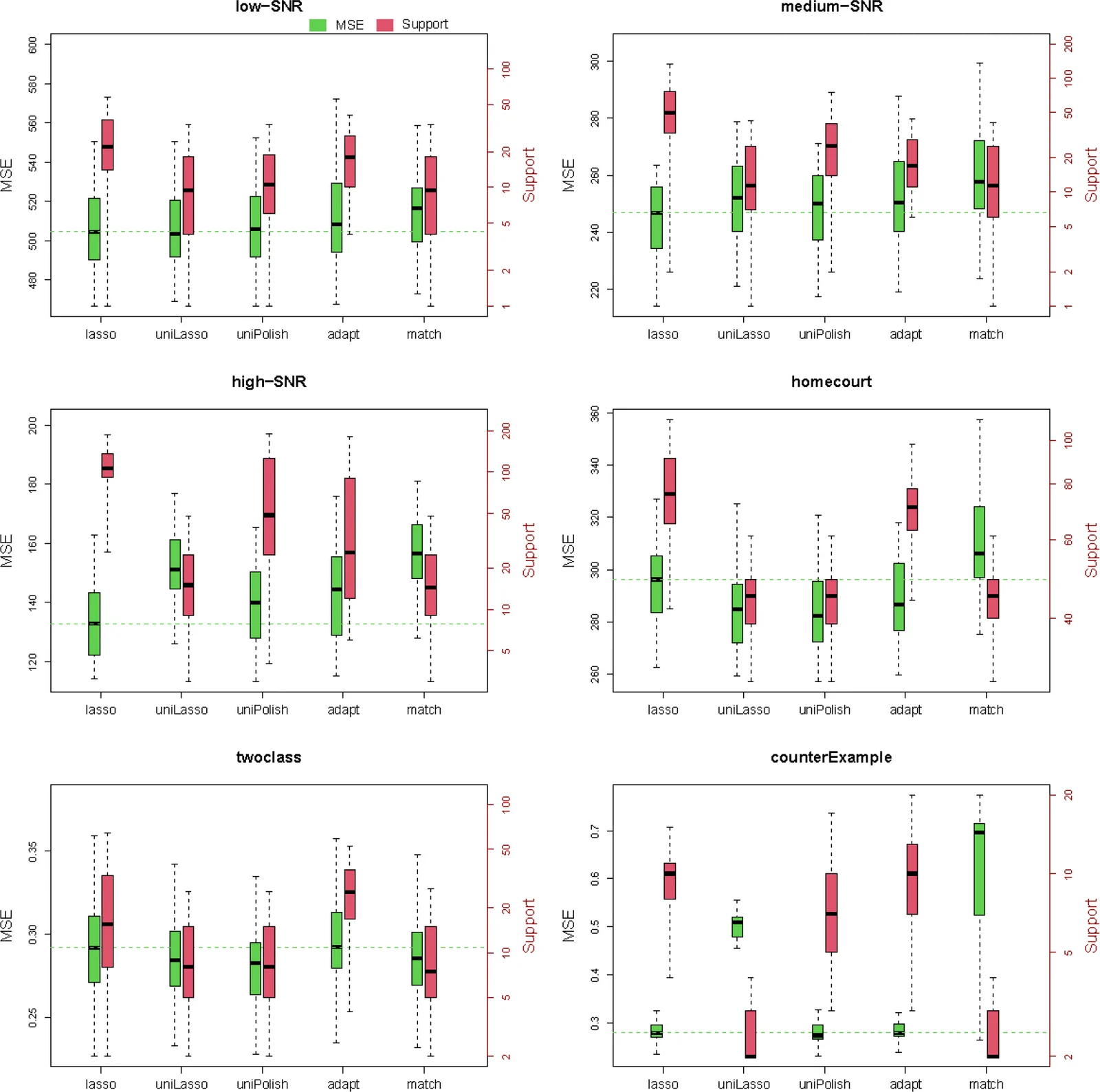
Test error (Gaussian) or misclassification rate (Binomial), along with support size. N=300,p=1000.

Figure 6 shows the results of a comparative study of uniLasso with lasso and three other sparse regression methods. Shown are means and standard errors over 100 simulations. The methods are:

- Lasso (using glmnet).
- UniLasso
- Polish: A postprocessing of the uniLasso solution, described in Section 11.3.
- Adaptive lasso using penalty weights $1/\left|{\hat{\beta }}_{j}\right|$ using the univariate coefficients ${\hat{\beta }}_{j}$. These first four methods use CV to estimate the value of λ that minimizes test error.
- Matching: A variant of the lasso, where we increase the λ parameter from the CV-based optimal value, until the support size matches that of uniLasso.

The simulation scenarios are:

1. **Low, medium, and high SNR**: Here n=300,p=1000,N(0,1) features with pairwise correlation 0.5. The nonzero regression coefficients are N(0,1); Gaussian errors with SDσ chosen to produce low < 1, medium (≈ 1), or high (> 2) SNRs.
2. **Homecourt**: Here n=100,p=30 with details as specified in Section 2.3.
3. **Two-class**: We set n=200,p=500 with a binary target y. The feature covariance within each class is AR(1) with ρ=0.8, and the first 20 features are shifted in the y=1 class by 0.5 units.
4. **Counter-example**: $n=100,\;p=20,\;x_{1}∼N(0,1)$, $x_{2}=x_{1}+N(0,1)$, β=(1,-.5,0,0,…0), error SD=0.5.

In the first three scenarios, 10% of the true coefficients are nonzero; for example, in (1), 100 of the 1,000 true coefficients are nonzero. Figure 6 shows the test set error (relative to the lasso) and support size (number of nonzero coefficients).

**Figure 7.**
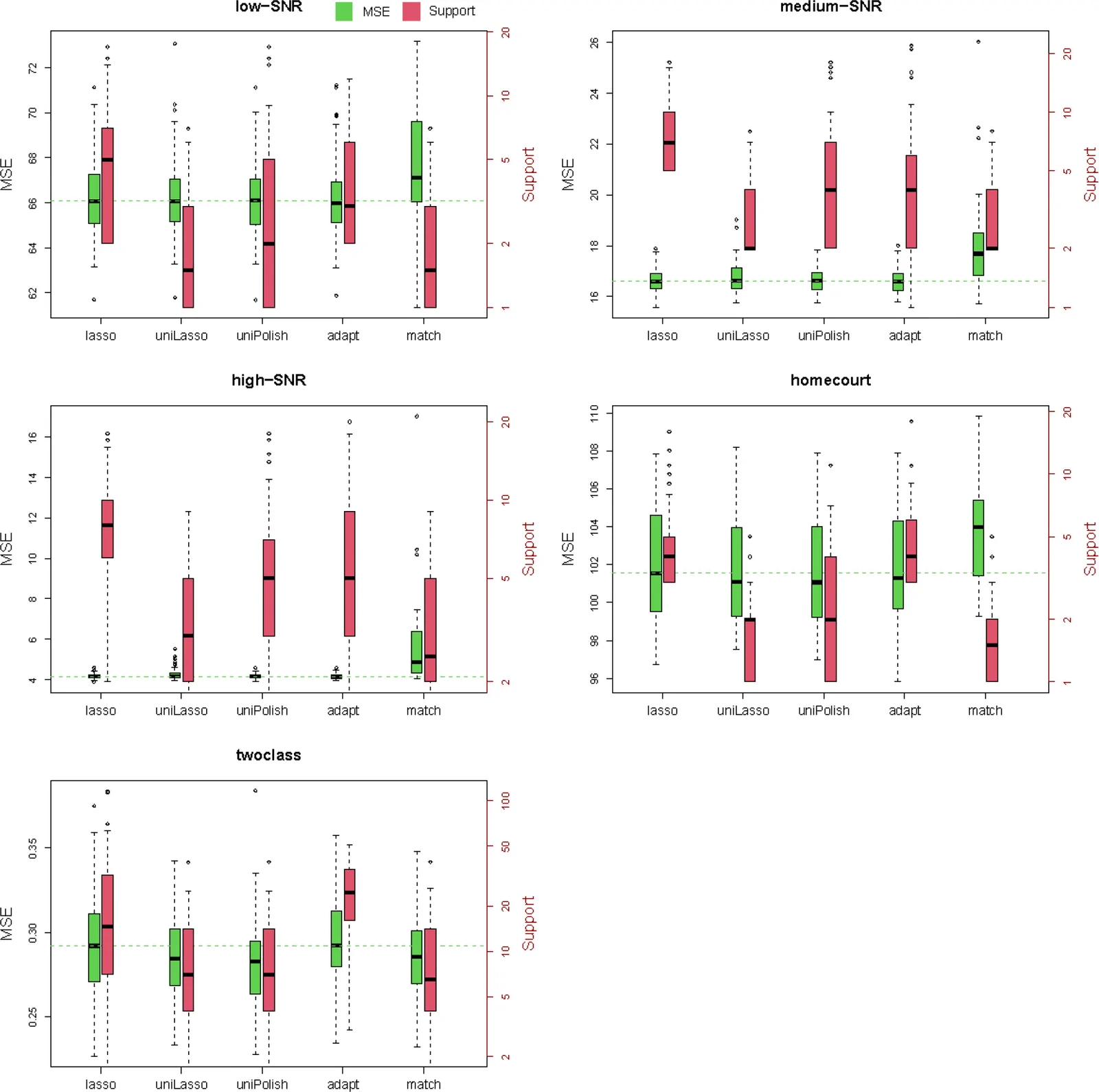
Test error (Gaussian) or misclassification rate (Binomial), along with support size. N=300,p=100.

Overall we see that uniLasso shows MSE similar to that of lasso, with often a much smaller support. The same general pattern holds with the real data sets. Exceptions to this are the high-SNR setting where lasso wins handily and the homecourt setting designed to exploit uniLasso’s strength (recall Theorem 2). The ‘counter-example’ setting is the classic case where features have positive correlation but the true regression coefficients have opposite signs; not surprisingly, uniLasso does poorly. The polish method and adaptive lasso do reasonably well, while the matching method does poorly. Of course in practice one has cross-validation to help determine which method is preferred in a given example. Figure 7 shows a similar pattern for the N=300,p=100 setting.

## 9. The Unregularized Setting: uniReg

Here we consider the case where λ=0, so that there is no $\ell_{1}$ penalty, as detailed in (2.1). While the main use case has n>p, we note that uniReg can be defined for p>n by taking λ→0 in uniLasso. Computationally, this will approximately give the solution with minimum $\ell_{1}$ norm, in the same way that the limiting lasso fit gives the least squares solution with minimum $\ell_{1}$ norm. Conveniently even without the $\ell_{1}$ penalty, the nonnegativity constraint still promotes sparsity in the solution.

In the next two sections we demonstrate the performance of uniReg on simulated and real data.

### 9.1. Simulated Data

In Table 5 we compare least squares with uniReg in a subset of the simulated settings defined in Section 8. UniReg performs remarkably well, often winning in MSE and yielding consistently sparser models.

### 9.2. Real Data

**Figure 8.**
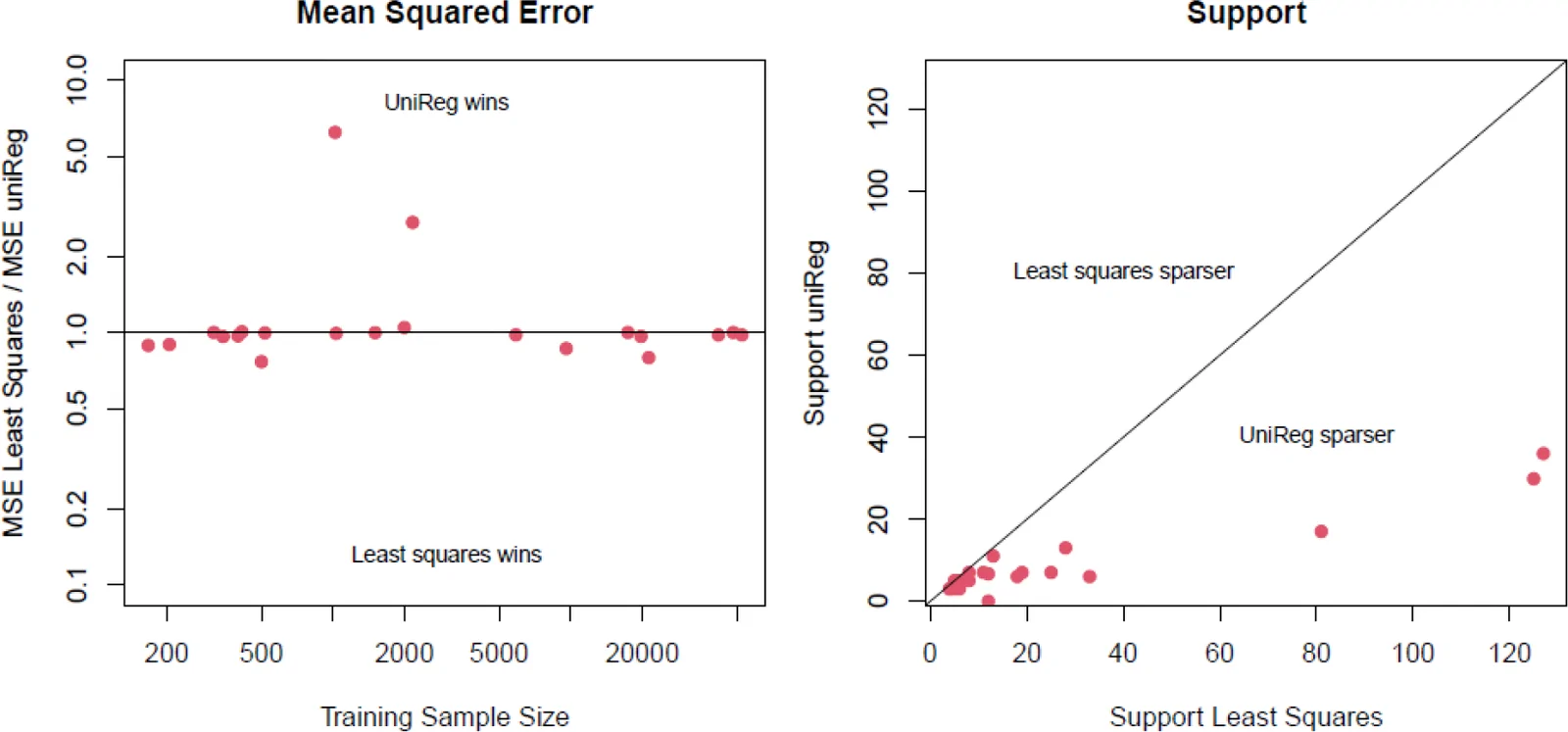
Least squares (LS) versus univariate regression model (uniReg) on regression data sets from the University of California, Irvine database. All data sets have n > p. The left plot shows the mean squared error (MSE) ratio on the log scale. The right panel shows the support sizes of the chosen models.

We compared least squares and uniReg on the regression data sets from the University of California, Irvine database.^4^ There were 23 data sets in all. Some had multiple targets: we treated each target separately and averaged the results. We randomly sampled each data set into a 70/30 training/test split, and report the test set MSE and support in Figure 8. One data set had a small sample (n=60) that produced a very large MSE from least squares; we omit this from the plot for visibility.

The two methods perform similarly in MSE; but uniReg has much smaller support, averaging about 1/3 that of the least squares support (which is the number of features p).

### 9.3. Theory for uniReg Without LOO

Our data consists of $\left(X_{i},Y_{i}\right),\;i=1,\;\ldots \;,n$, where

$$Y_{i}=\beta_{0}+X_{i}^{T}\beta +\epsilon_{i},$$

where $\beta_{0}\in R,\;\beta =\left(\beta_{1},\;\ldots \;,\beta_{p}\right)\in R^{p},\;X_{i}$ are independent and identically distributed $N_{p}(0,\Sigma )$ random vectors, where Σ is a positive definite matrix with minimum eigenvalue $\lambda_{0}$ and maximum eigenvalue $\lambda_{1}$, and $\epsilon_{i}$ are independent and identically distributed $N\left(0,\sigma^{2}\right)$ random variables for some σ>0. Let r:=p/n. We assume that $r\in \left(0,r_{0}\right)$ for some $r_{0}<1$.

Let ${\hat{\beta }}_{0}^{\text{OLS}}$ and ${\hat{\beta }}^{\text{OLS}}$ denote the ordinary least squares (OLS) estimates of $\beta_{0}$ and β. Let ${\hat{\beta }}_{0}^{\text{UR}}$ and ${\hat{\beta }}^{UR}$ denote the uniReg estimates of $\beta_{0}$ and β *without leave-one-out*, defined as follows. First, compute the univariate coefficients ${\hat{\beta }}_{j}^{\mathrm{uni}},\;j=1,\;\ldots \;,p$, as the coefficient of $X_{j}$ when Y is regressed solely on $X_{j}$ with an intercept term. The estimates ${\hat{\beta }}_{0}^{UR}$ and ${\hat{\beta }}^{UR}$ are obtained by minimizing

$$\sum_{i=1}^{n}{\left(Y_{i}-a-X_{i}^{T}b\right)}^{2}$$

over a∈R and $b\in R^{p}$ subject to the constraint that $\beta_{j}{\hat{\beta }}_{j}^{\mathrm{uni}}\geq 0$ for j=1,…,p. Let $\beta_{0,j}^{\mathrm{uni}}$ and $\beta_{j}^{\mathrm{uni}}$ be the population univariate coefficients; that is,

$$E\left(Y_{i}|X_{i,j}\right)=\beta_{0,j}^{\mathrm{uni}\;}+\beta_{j}^{\mathrm{uni}\;}X_{i,j}.$$

Define three vectors $\mu ,{\hat{\mu }}^{OLS},{\hat{\mu }}^{UR}\in R^{n}$ as

$$\mu_{i}:=E\left(Y_{i}|X_{i}\right)=\beta_{0}+X_{i}^{T}\beta ,$$

$${\hat{\mu }}_{i}^{OLS}:={\hat{\beta }}_{0}^{OLS}+X_{i}^{T}{\hat{\beta }}^{OLS},$$

$${\hat{\mu }}_{i}^{UR}:={\hat{\alpha }}_{0}^{UR}+X_{i}^{T}{\hat{\alpha }}^{UR}$$

for i=1,…,n. Let q be the number of j such that $\beta_{j}=0$ and $\beta_{j}^{\mathrm{uni}}\neq 0$. The following theorem shows that if $\beta_{j}$ and $\beta_{j}^{\mathrm{uni}}$ have the same sign for all j, then $E{\left\|\mu -{\hat{\mu }}^{UR}\right\|}^{2}$ is less than or equal to $E{\left\|\mu -{\overset{ˆ}{\mu }}^{OLS}\right\|}^{2}$ minus a constant times q.

#### Theorem 4.

*Suppose that* $\beta_{j}^{\mathrm{uni}}\beta_{j}\geq 0$ *for each* 1≤j≤p. Let δ *be the minimum of* $\left|\beta_{j}^{\mathrm{uni}}\right|$ *over* 1≤j≤p *such that* $\beta_{j}^{\mathrm{uni}}\neq 0$*. Then there are positive constants* $C_{0},\;C_{1}$ *depending only on* $\lambda_{0},\;\lambda_{1},\;\delta ,\;r_{0}$, *and* σ *such that if* $n\geq C_{0}$, *then*

$$E{\left\|\mu -{\overset{ˆ}{\mu }}^{UR}\right\|}^{2}\leq E{\left\|\mu -{\overset{ˆ}{\mu }}^{OLS}\right\|}^{2}-C_{1}q.$$

The theorem is proved in Appendix D. We have not been able to prove the LOO version of uniReg (that is, the actual version that we have proposed). We leave it as an open question.

## 10. Real Data Examples for uniLasso

**Figure 9.**
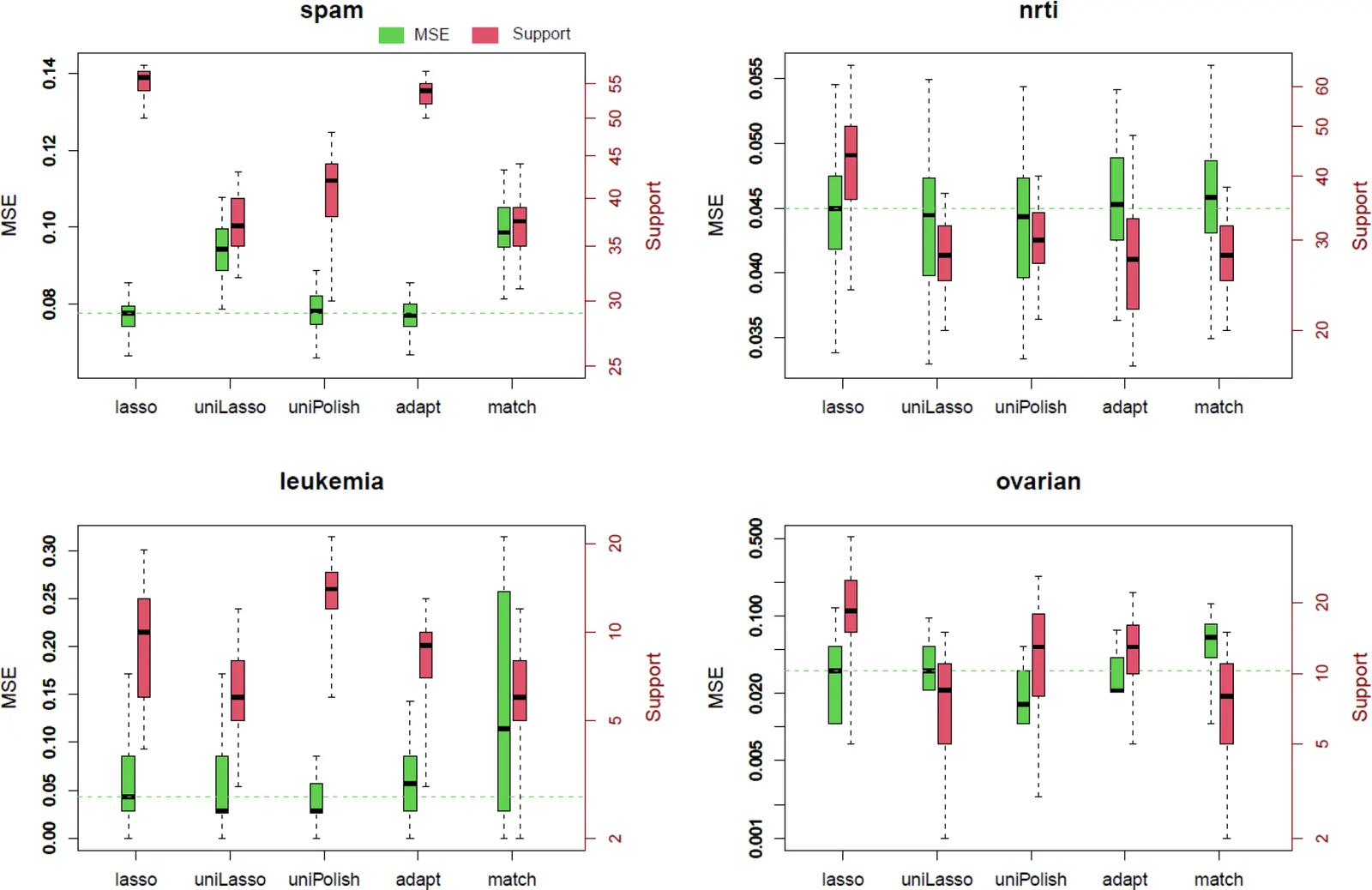
Real data examples: Blue lines: relative test error (Gaussian) misclassification rate (binomial) or deviance (Cox), with support size superimposed in gold. MSE = mean squared error.

Table 6 gives a high level summary of six data sets that we examined in this study. Figure 9 shows the results: these are test error and support size averaged over 100 train/test 50–50 splits.

We see the same general trend as in the simulated examples: uniLasso tends to give test error similar to that of lasso, with often smaller support size.

## 11. Properties, Extensions, and Computation of the Proposed Method

### 11.1. Multiclass Models

Here we consider a classification problem with more than two classes. The multinomial model is natural for the multiclass problem, but does not generalize easily to uniLasso. The reason is that for each feature there are coefficients for each class, so it is not clear how to replace the feature with a LOO version. Furthermore the coefficients per class are only determined up to a shift, and hence a nonnegativity constraint does not make sense. Instead we take a ‘one-versus rest’ (OVR) approach, where the uniLasso binary classifier is used to classify each class from the others. Hence each class gets a predicted probability, and with OVR one classifies to the class with the highest probability as the predicted class.

We applied this to a data set on childhood cancer, with 63 training and 25 test samples in four classes (n.d.-n). With the original train/test split we get zero test errors with glmnet. We repeated the train/test split 50 times and obtained the results shown in Table 7.

### 11.2. The Use of External Univariate Scores

Consider the setting where we have our training set T and also external data E from the same domain (e.g., disease), We assume that E does not contain raw data but only summary results. Specifically, E contains just univariate coefficients and standard errors for each feature. This setting occurs fairly often in biomedical settings where investigators are not willing to share their raw data, but do publish and share summary results.

We demonstrate here how uniLasso can make productive use of external scores. The idea is to use the univariate coefficients from E, rather than computing the LOO estimates from the training set. Then in Step 2 of uniLasso, we proceed as usual.

**Figure 10.**
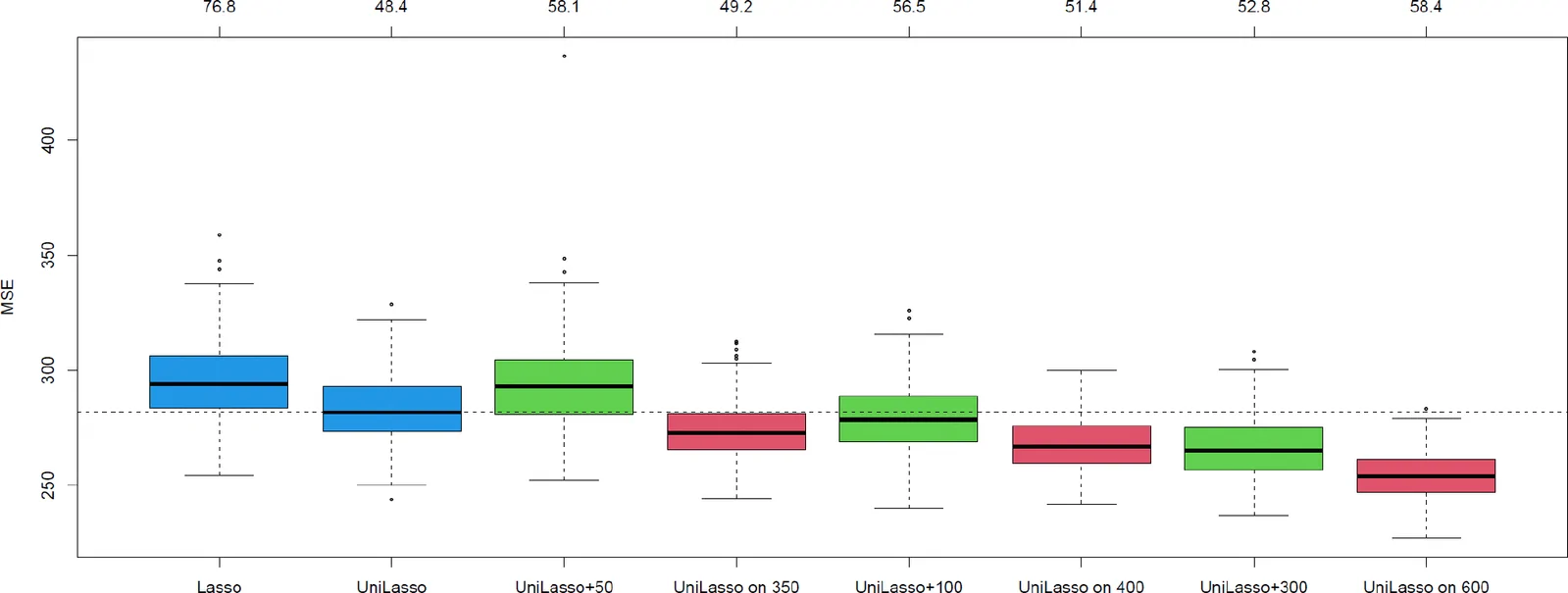
Results from an experiment examining the use of external data with uniLasso. The initial training set has 300 observations and we generated 600 additional samples. Shown in blue are the test set MSE for the lasso, and uniLasso, both applied just to the training set; uniLasso + m (green), where the univariate scores derived from the m extra samples are used in place of the LOO estimates, and uniLasso (red) on 300+m samples for m=50,100,...300. The support size of each model is shown along the top margin.

To investigate this scheme, we generated data T with n=300,p=1000,SNR=1.5, with feature covariance AR(1), with ρ=0.8 and the non-zero regression coefficients distributed as U(0.5,2), and positioned on features (1,3,5, … 99). We also generated 600 extra samples from the same distribution to serve as an external data set E, and in addition a large test set. Figure 10 shows the test-set MSE over 200 simulations from the following strategies:

1. lasso and uniLasso, both applied just to the training set;
2. uniLasso+ m (green) where the univariate scores are derived from m extra samples from E, and are used in place of the LOO estimates;
3. uniLasso (red) on all 300+m samples.

We see that adding the scores from just 50 external observations does slightly worse than uniLasso on the original data, but builds sparser models than lasso at no cost in test MSE. If instead, we use the extra 50 samples as in strategy 3, the performance improves. As we increase m further, both strategies 2 and 3 outperform lasso and uniLasso both fit on only the original 300 observations. Strategy 3 is best, but if we have only the raw external scores, strategy 2 is the method of choice. We note that the strategy 2 decreases the sparsity of uniLasso a moderate amount.

This is one simulation scenario, and it is of course possible that in others the pattern may not be as clear.

### 11.3. A uniLasso Polish

As we have seen earlier, the uniLasso procedure can sometimes perform considerably worse than the lasso, especially in cases where its sign constraint is too restrictive. An example is the ‘counter-example’ problem in Section 8, where two positively correlated features have different coefficient signs in the population multivariate model. While problematic, the CV estimate of error will alert the user that uniLasso is not working well in a given problem. In this section, we propose a simple ‘postprocessing’ of the uniLasso solution that can help to diagnose shortcomings of uniLasso and remedy them.

The idea is very simple: we run the usual lasso (with cross-validation) to the residual from the uniLasso fit, and use this to extend the solution path of the uniLasso fit. Here is the algorithm in detail, using the glmnet R package terminology:

1. Run cv.uniLasso on X,y, and extract the fitted linear predictor $\hat{y}$ at ${\hat{\lambda }}_{\min}$.
2. Apply cv.glmnet on X,y, with offset $\hat{y}$, giving final predictions ${\hat{y}}_{g}$.
3. Use this to extend the original uniLasso path, and choose a solution via cross-validation.

Note that in the Gaussian model the use of the offset is equivalent to applying the lasso to the residual in Step 2; expressed as an offset it allows application of the idea to other GLM families.

**Figure 11.**
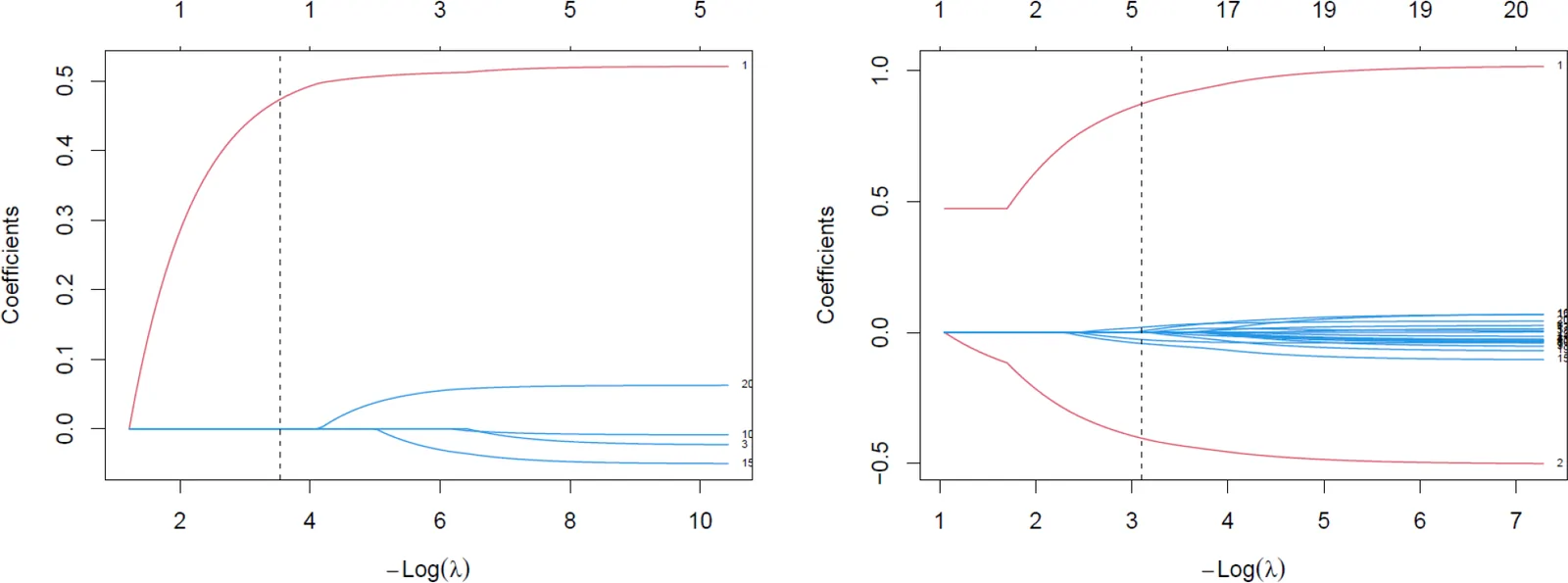
UniLasso polish applied to the counter-example problem of Section 8. The left panel shows the uniLassouniLasso solution path, with the dashed vertical line indicating the solution chosen by cross-validation. The right panel shows the polish.uniLassopolish.uniLasso solution path, which starts at the chosen uniLassouniLasso solution, and the vertical dashed line indicates the model chosen by cross-validation. The red curves are the support features $x_{1}\times 1$ and $x_{2}\times 2$.

Figure 11 shows the results of the uniLasso polish applied to the counter-example problem of Section 8. There are two support features ($x_{1}$ and $x_{2}$). In the left panel uniLasso enters just feature $x_{1}$, because $x_{2}$ has a positive univariate coefficient but a negative multivariate coefficient. The right panel shows the 'polished' path. Via the offset it starts at the uniLasso solution (chosen by CV), and immediately enters $x_{2}$, and eventually growing the coefficients of both $x_{1}$ and $x_{2}$. In the process, the model lets in some of the noise variables. In Section 8 we included the uniLasso polish in all of the settings, and its performance overall is excellent.

### 11.4. Does CV Work in Our Two-Stage uniLasso Procedure?

In the uniLasso algorithm we use the target y in both steps, but for (significant) computational convenience, we only apply cross-validation in the second step. Thus one should be concerned that cross-validation may not perform well here. In the simulations of Section 8 we used this form of cross-validation to choose the λ tuning parameter, and it seems to have done a reasonably good job.

**Figure 12.**
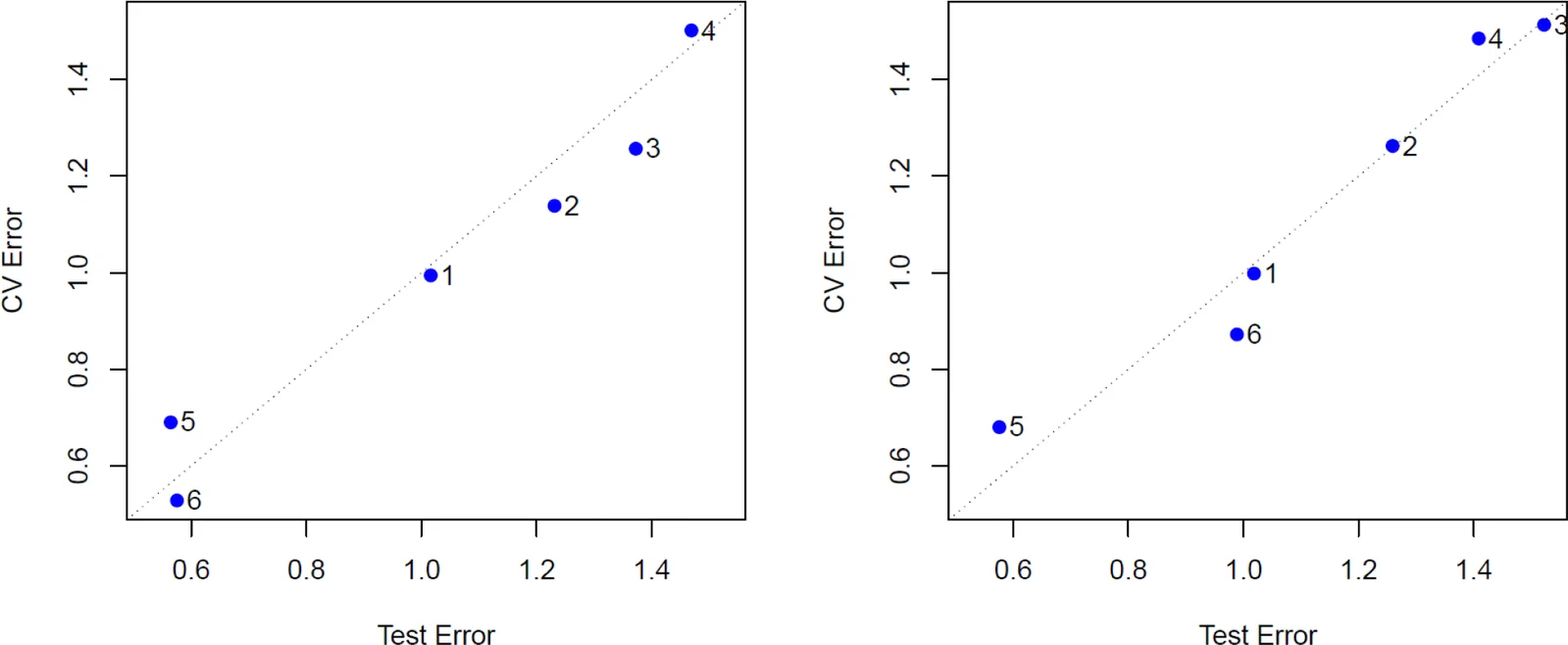
Cross-validation (CV) error at the selected value of λ versus the test error, for the lasso (left) and uniLasso (right). Each datapoint represents one of our simulated data sets with p>N studied earlier. Both CV estimates track the test error reasonably well.

A related question is whether the error reported by CV is a good estimate of test error, especially at the selected value of λ. Figure 12 shows the CV error at the selected value of λ versus the test error, for the lasso (left) and uniLasso (right). We see that both CV estimates are quite good. It seems clear that our use of cross-validation in uniLasso is not a major concern.

**Figure 13.**
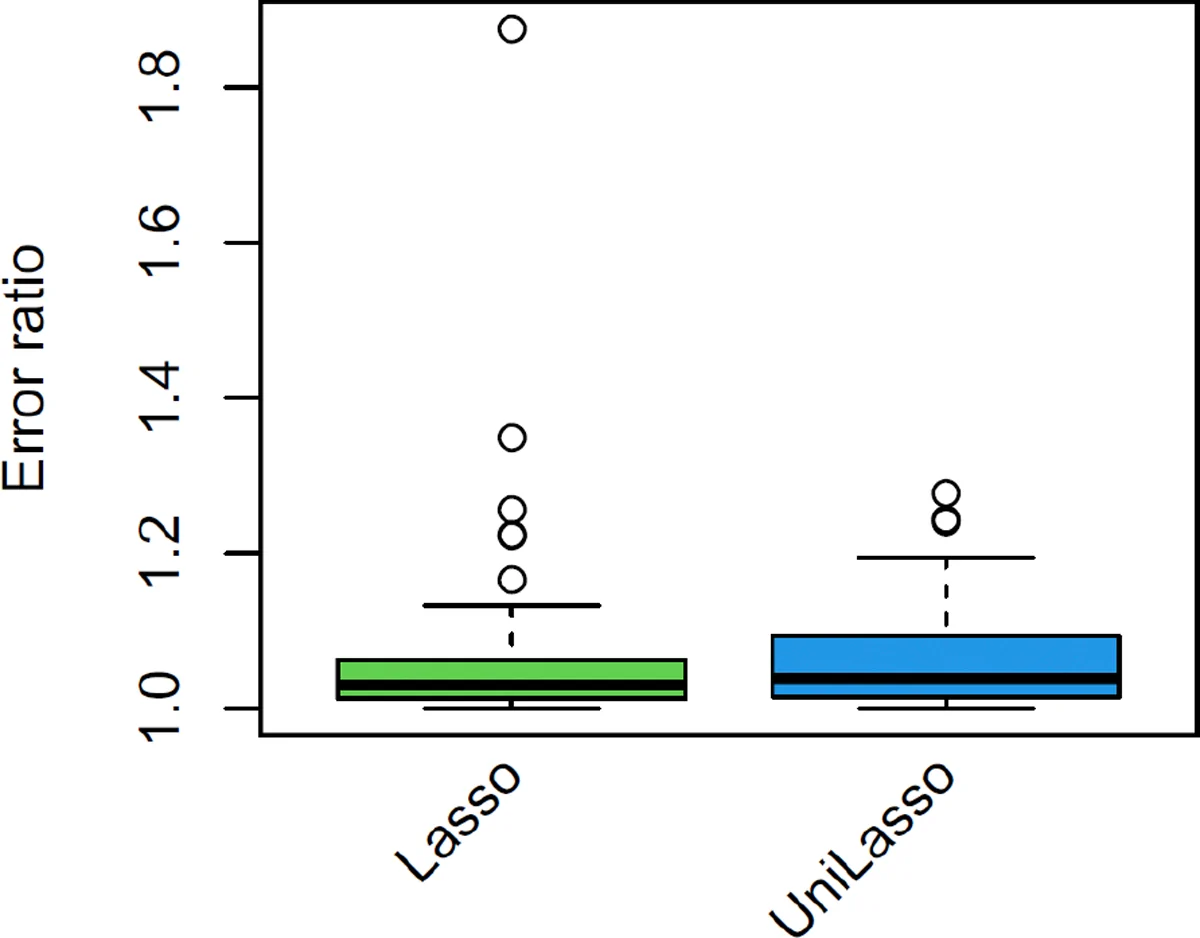
Car price data: ratio of the achieved test error using cross-validation (CV) divided by the minimum test error over the λ path.

Finally, a referee asked whether a reason for uniLasso’s strong performance was its ability to estimate the CV tuning parameter λ. For the car price data, Figure 13 shows the ratio of the achieved test error using CV divided by the minimum test error over the λ path, for each of the 50 train/test splits. We see that both lasso and uniLasso do an (equally) good job of estimating the optimal λ. The average ratios between the λ chosen by cross-validation and the optimal choice was 0.87 and 0.38 for the lasso and uniLasso, respectively.

### 11.5. UniLasso for GLMs and the Cox Survival Model

Our discussion of uniLasso has focused on squared-error loss. However, we can use the same idea in any scenario where we are able to fit a lasso model and produce a scalar prediction function η(x). This includes in particular all generalized linear models, and the Cox proportional hazards model. These models are included in glmnet.

The steps are almost the same as before (we will discuss for GLMs):

1. Fit the p univariate GLMS, and produce the linear predictor functions ${\hat{\eta }}_{j}^{i}={\hat{\beta }}_{0j}+{\hat{\beta }}_{j}x_{ij}$. Compute the LOO predictions ${\hat{\eta }}_{j}^{-i}$ for the n training observations for each of these.
2. Using these LOO predictions as features, fit a non-negative lasso GLM to the response (using CV for selection of λ), yielding coefficients $\hat{\theta }={\left({\hat{\theta }}_{0},{\hat{\theta }}_{1},\cdots ,{\hat{\theta }}_{p}\right)}^{⊤}$.
3. Return the composite estimated linear predictor

(11.1) $$\hat{\eta }(x)={\hat{\theta }}_{0}+\sum_{j=1}^{p}{\hat{\theta }}_{j}{\hat{\eta }}_{j}\left(x_{j}\right),$$

which collapses as before to a linear model $\hat{\eta }(x)={\hat{\gamma }}_{0}+\sum_{j=1}^{p}{\hat{\gamma }}_{j}x_{j}$.

Step 1 can be computationally challenging. Estimating the p univariate functions ${\hat{\eta }}_{j}\left(x_{j}\right)$ is fairly straightforward, since each require a few very simple Newton steps. The LOO estimates are more challenging. While for squared-error loss, we have simple formulas for computing these exactly, this is not the case for nonlinear models. However, there are good approximations available that are computationally manageable. We discuss all the computational aspects in the next section.

### 11.6. Efficient Computations for uniLasso

#### 11.6.1. Least Squares

We first discuss the computations for squared-error loss. For feature j we have to fit a univariate linear model, and then compute its LOO predictions. We are required to fit the model $\eta_{j}\left(x_{j}\right)=\beta_{0j}+\beta_{j}x_{j}$ using the data ${\left\{x_{ij},y_{i}\right\}}_{1}^{n}$. This task is simplified if we standardized $x_{ij}$ to have mean zero and unit variance: $z_{ij}=\left(x_{ij}-{\bar{x}}_{J}\right)/s_{j}$, where ${\bar{x}}_{j}=\frac{1}{n}\sum_{i=1}^{n}x_{ij}$, and $s_{j}^{2}=\frac{1}{n}\sum_{i=1}^{n}{\left(x_{ij}-{\bar{x}}_{j}\right)}^{2}$. Then the least squares estimates using the $z_{ij}$ are $\left({\hat{\delta }}_{0j},{\hat{\delta }}_{j}\right)=\left(\bar{y},\frac{1}{n}\sum_{i=1}^{n}z_{ij}y_{i}\right)$. These are mapped back to least squares estimates for $x_{ij}$ via $\left({\hat{\beta }}_{0j},{\hat{\beta }}_{j}\right)=\left({\hat{\delta }}_{oj}-{\hat{\delta }}_{j}{\bar{x}}_{j}/s_{j},{\hat{\delta }}_{j}/s_{j}\right)$.

This standardized form is particularly useful for computing the LOO fits, since the fits are invariant under affine transformations of the features (so we can use the $z_{ij}$ instead of the $x_{ij}$). We have the classical formula for the LOO residual in linear regression

(11.2) $$y_{i}-{\hat{\eta }}_{j}^{-i}=\frac{y_{i}\;-\;{\hat{\eta }}_{j}^{i}}{1\;-\;H_{ii}},$$

where $H_{ii}$ is the ith diagonal entry of the *hat* matrix. It is straightforward to show that using the $z_{ij}$ gives $H_{ii}=1/n+z_{ii}^{2}/n$. Now simple algebra backs out an expression for ${\hat{\eta }}_{j}^{-i}$.

These operations can be performed efficiently for all i and p simultaneously using matrix operations in R, without the need for any loops. In particular we can perform *Hadamard* (elementwise) arithmetic operations (e.g., multiplication) of like-sized matrices in single operations.

#### 11.6.2. Generalized Linear Models

The discussion here also applies to the Cox model. Fitting a GLM by maximum likelihood is typically done using a Newton algorithm. This iterative algorithm amounts to making a quadratic approximation to the negative log-likelihood at the current solution, and then solving the quadratic problem to get the updated solution. For GLMs this can be cast as *iteratively reweighted least squares* (IRLS).

Given the fitted linear predictor vector $\eta_{j}^{\left(\ell \right)}$ at iteration ℓ, one forms a working response vector $z_{j}^{\left(\ell \right)}$ that depends on $\eta_{j}^{\left(\ell \right)}$ and y and other properties of the particular GLM family, and an observation weight vector $w_{j}^{\left(\ell \right)}$, and fits an updated model $\eta_{j}^{\left(\ell +1\right)}$ by weighted least squares of $z_{j}^{\left(\ell \right)}$ on $x_{j}$ with weights $w_{j}^{\left(\ell \right)}$. Usually four iterations are sufficient.

Once again we can fit all the univariate GLMs using matrix operations at the same time—that is, we perform each IRLS step simultaneously for all p univariate GLMS. The expressions are only slightly more complex than in the unweighted case.

What about the LOO fits ${\hat{\eta }}_{j}^{-i}$ for each univariate GLM? Here we make an approximation, as recommended by (n.d.-o), which amounts to using the final weighted least squares IRLS iteration when fitting the models ${\hat{\eta }}_{j}\left(x_{j}\right)$. A simple formula is available there, similar to what we got before, except a bit more detailed to accommodate observation weights. Again we can use Hadamard matrix operations to do this simultaneously for all i and j.

For the Cox model, we make a further approximation, since the implied eight matrix in IRLS is not diagonal. We simply use the diagonal, which leads to an approximate Newton algorithm. Note that for the Cox model the intercept is always zero.

#### 11.6.3. Software in R

We provide an R package uniLasso for fitting the models described in this article. It mirrors the behavior of the glmnet package for fitting lasso models. The function cv.uniLasso has arguments that include all those for cv.glmnet plus a few extras. This function does the following, currently for “binomial,” “gaussian,” and “cox” families. It does all the work outlined in Sections 11.6.1–11.6.2, and performs the second stage cv.glmnet using the (approximate) LOO ${\hat{\eta }}_{j}^{-i}$ as features. A cv.glmnet object contains information about suggested values of λ, as well as a fitted glmnet model fit to all the data with solutions at all values of λ. This object is referenced when one predicts from a cv.glmnet object. In this case we make sure that the coefficients that are stored on this objects for each λ are the collapsed versions as in (11.1).

Hence cv.uniLasso returns a bona fide cv.glmnet object, for which there are a number of methods for plotting, printing, and making predictions.

cv.uniLasso has three important arguments:

1. loo = TRUE: by default it uses the ${\hat{\eta }}_{j}^{-i}$ as features. If this is set to FALSE it will use the univariate fitted values ${\hat{\eta }}_{j}^{i}$ instead.
2. lower.limits = 0: this default choice guarantees that the ${\hat{\theta }}_{j}$ are all nonnegative. We recommend this in order to get a sparser and more interpretable model.
3. standardize = FALSE: this default is the most sensible to use, since part of the point here is to boost strong variables through their scale. Standardizing would undo that.

In addition we have uniReg and cv.uniReg for fitting the uniReg model, as well as ci.uniReg for computing bootstrap intervals for each coefficient. The function polish.uniLasso does the polishing as described in Section 11.3. We also provide simulation functions for generating the data as used in this paper.

## 12. Discussion

In this article we have introduced a novel method for sparse regression that ‘stacks’ univariate regressions using a nonnegative version of the lasso. The procedure has interesting properties, namely, that the final features weights have the same signs as the univariate coefficients and tend to be larger when the univariate coefficients are large. The test-set MSE of the method is often similar to that of the lasso, with substantially smaller support and lower false positive rate.

There are many possible extensions for this work, for example, to random forests, gradient boosting, and neural networks. These are topics for future study.

## Figures and Tables

**Table 1.** **Proteomics study. Top table:** Univariate least squares (LS) coefficients and lasso coefficients, for model chosen by lasso. The two sign changes from univariate to multivariate are shown in blue. **Bottom table:** same for model chosen by uniLasso. Biomarkers are ordered by decreasing absolute value of the univariate coefficients. Most biomarkers are different in the two tables, with the exception of 1 and 3 in the first table corresponding to H and I in the second table.

Model chosen by lasso		

Biomarker	Univariate LS Coefficient	Lasso Coefficient
1	−49.24	−18.92
2	−37.65	−5.42
3	25.27	37.49
4	−24.74	−11.26
5	22.91	14
6	−22.38	−12.75
7	−17.97	−11.91
8	13.02	0.79
9	−12.71	−1.85
10	−10.14	−2.32
11	1.81	−6.27
12	−0.34	6.25
Model chosen by uniLasso		
Biomarker	Univariate LS Coefficient	uniLasso Coefficient
A	93.13	13.82
B	−84.26	−8.17
C	77.69	15.86
D	73.84	27.66
E	69.15	16.21
F	58.92	1.38
G	−53.46	−10.5
H	−49.24	−20.76
I	25.27	10.48

**Table 2.** Test MSE (mean squared error), support, TPR (true positive rate), and FPR (false positive rate) for 100 simulations from the homecourt setting.

	MSE-lasso	MSE-uniLasso	Support-lasso	Support-uniLasso

Mean	1.098	1.077	7.660	4.790
SE	0.005	0.005	0.309	0.142
	TPR-lasso	TPR-uniLasso	FPR-lasso	FPR-uniLasso
Mean	0.737	0.700	0.135	0.025
SE	0.014	0.016	0.012	0.004

**Table 3.** Results for 50 simulations from the medium-SNR setting. The last two columns use variations of uniLasso: noSign removes the nonnegative constraint, while noMag uses just the sign of the univariate estimates in Step 2 of uniLasso (not the magnitude).

LOO				

	lasso	uniLasso	uniLasso-noSign	uniLasso-noMag
MSE	0.55	0.59	0.61	0.60
SE	0.03	0.03	0.04	0.04
Support	53.78	15.32	66.38	40.12
SE	4.41	1.19	12.92	6.47
No LOO				
	lasso	uniLasso	uniLasso-noSign	uniLasso-noMag
MSE	0.55	0.61	0.57	0.61
SE	0.03	0.04	0.03	0.05
Support	53.78	36.84	24.48	48.98
SE	4.41	6.34	2.71	9.66

*Note*. LOO = leave-one-out. MSE = mean squared error. SNR = signal-to-noise ratio.

**Table 4.** Coefficients from various models fit to the car price data. Coefficients with sign changes relative to the univariate fits are marked in blue.

	Univariate	Lasso	UniLasso	Polish

body.style-3	−4.768	−0.224	0.000	0.000
wheel.base	0.781	0.035	0.000	0.000
width	3.082	0.475	0.378	0.000
height	0.326	−0.154	0.000	0.000
curb.weight	0.014	0.004	0.002	−0.001
engine.type-7	−0.652	3.303	0.000	0.000
num.of.cylinders-2	9.867	−2.546	0.000	0.000
num.of.cylinders-3	−13.854	0.000	−0.545	0.000
num.of.cylinders-4	10.543	−0.667	0.000	0.000
num.of.cylinders-7	−0.652	0.009	0.000	0.000
engine.size	0.167	0.064	0.048	0.000
fuel.system-2	−9.143	0.208	0.000	0.000
fuel.system-5	−0.694	−1.248	0.000	0.000
bore	17.571	−3.729	0.000	0.000
horsepower	0.169	0.017	0.042	0.000
peak.rpm	−0.001	0.000	0.000	0.001

**Table 5.** Comparison of least squares with uniReg in a subset of simulated settings defined earlier.

*N* = 300, *p* = 30						

Setting	*p*	SNR	MSE-LS	MSE-uniReg	Supp-LS	Supp-uniReg
low SNR	30	0.092	71.339	65.171	30	4
medium SNR	30	0.369	17.836	16.489	30	4
high SNR	30	1.475	4.459	4.178	30	4
homecourt	30	0.059	111.771	101.542	30	4
*N* = 300, *p* = 100						
Setting	*p*	SNR	MSE-LS	MSE-uniReg	Supp-LS	Supp-uniReg
low SNR	100	0.318	97.678	69.655	100	8
medium SNR	100	1.272	24.428	18.559	100	9
high SNR	100	5.087	6.104	5.82	100	7
homecourt	100	0.228	149.683	104.979	100	13
*N* = 300, *p* = 1,000						
Setting	*p*	SNR	MSE-LS	MSE-uniReg	Supp-LS	Supp-uniReg
low SNR	1,000	0.578	842.191	510.837	300	24.5
medium SNR	1,000	1.48	374.915	249.076	300	27
high SNR	1,000	3.613	186.171	147.276	300	27
homecourt	1,000	1.53	577.852	420.508	300	135.5

*Note*. SNR = signal-to-noise ratio. MSE-LS = mean squared error for least squares. MSE-uniReg = mean squared error for uniReg. Supp-LS = support of least squares. Supp-uniReg = support of the uniReg model.

**Table 6:** Summary of data sets used in our study.

Data set	*n*	*p*	Feature type	Outcome type

Spam	2,301	57	mixed	binary
NRTI	1,005	211	binary mutations	drug effectiveness (quantitative)
Leukemia	72	7,129	gene expression (quantitative)	binary
DLBCL	240	1,000	gene expression (quantitative)	survival
Breast Cancer	157	1,000	gene expression (quantitative)	survival
Ovarian	190	2,238	Mass spec peak intensities	binary

*Note*. NRTI = nucleoside reverse transcriptase inhibitor. DLBCL = diffuse large B-cell lymphoma.

**Table 7.** Results for the four-class cancer example. Overall uniLasso one-versus rest (OVR) makes the fewest misclassification errors averaged over the 50 train-test splits. Of the 50 train(63)/test(25) splits, 41 made zero errors on the 25 test data points, and 9 made 1 error, averaging 0.18. Lasso OVR is sligthly worse, and lasso multinomial makes almost one error on average per train/test split. On the other hand lasso multinomial yields the sparsest model, since the other two select features separately for each class.

	Ave # Errors	*SD*	Ave Support	*SD*

Lasso multinomial	0.96	0.03	18.68	0.08
UniLasso OVR	0.18	0.01	27.02	0.05
LassoOVR	0.22	0.01	58.08	0.08

# of Errors	0	1	2	3	4	7

Lasso multinomial	27	9	8	4	1	1
UniLasso OVR	41	9				
Lasso OVR	39	11				

## Footnotes

1 In this case we could simply scale the features by their univariate slope coefficients and ignore the intercepts, which would be handled by the overall intercept in the model.

2 We thank Chris Harbron for this analysis.

3 In the extreme case of zero correlation between yy and the jjth feature, the univariate fit is ${\hat{\beta }}_{0j}=\bar{y}\beta^{0}\text{j}={\text{y}}^{-}$, and one can show that the correlation between yy and the LOO version of $\bar{y}\bar{y}$ is −1!

4
https://archive.ics.uci.edu/datasets

## Appendices

### Appendix A. Proof of Proposition 1

If you substitute $\eta_{j}^{i}={\hat{\beta }}_{0j}+{\hat{\beta }}_{j}x_{ij}$ in the objective in (3.2) you get

$$\sum_{i=1}^{n}{\left(y_{i}-\left(\theta_{0}+\sum_{j=1}^{p}{\hat{\beta }}_{0j}\theta_{j}\right)-\sum_{j=1}^{p}x_{ij}{\hat{\beta }}_{j}\theta_{j}\right)}^{2}+\lambda \sum_{j=1}^{p}\left|\theta_{j}\right|.$$

Now you change variables to $\gamma_{0}=\theta_{0}+\sum_{j=1}^{p}{\hat{\beta }}_{0j}\theta_{j}$ and $\gamma_{j}={\hat{\beta }}_{j}\theta_{j}$.

The result is immediate, since

$$\left|\theta_{j}\right|=\frac{\left|\gamma_{j}\right|}{\left|{\hat{\beta }}_{j}\right|}$$

and

$$\left\{sign\left(\gamma_{j}\right)=sign\left({\hat{\beta }}_{j}\right)\forall j\right\}\equiv \left\{\theta_{j}\geq 0\;\forall j\right\}$$

■

### Appendix B. Derivation Supporting Section 4

We wish to establish when the correlations of the univariate LOO fitted values with the response y are likely to be positive. Thanks to Chris Harbron for outlining this analysis.

Assume we have standardized both the feature vectors x_j and response y to have zero mean and unit variance. Since the analysis focuses on individual features, we will drop the index j.

Formula (11.2) relates the LOO residuals to the OLS residuals:

$$y_{i}-{\hat{\eta }}^{-i}=\frac{y_{i}\;-\;{\hat{\eta }}^{i}}{1\;-\;H_{ii}}.$$

Since both x and y are standardized, the vector of OLS fits is given by

(D.1)
$$\hat{\eta }\;=x\hat{\beta }\;\;=x\frac{x^{⊤}y}{n}$$

With $D=diag\left(\frac{1}{1-H_{11}},\frac{1}{1-H_{22}},\;\ldots \;,\frac{1}{1-H_{nn}}\right)$ we can write the vector of LOO fits as

(D.2)
$${\hat{\eta }}^{loo}=(I-D)y+Dx\left(x^{⊤}y\right)/n.$$

We look at the sample covariance between y and ${\hat{\eta }}^{\mathrm{loo}}$

(D.3)
$$Cov(y,{\hat{\eta }}^{loo})=1-\left(y^{⊤}Dy\right)/n+\left(y^{⊤}Dx\right)\left(x^{⊤}y\right)/n^{2}.$$

We would like to know when this covariance is positive. (Note that it is always nonnegative if we replace ${\hat{\eta }}^{loo}$ with $\hat{\eta }$). Since x is standardized,

$$H_{ii}=\frac{1}{n}+\frac{x_{i}^{2}}{n}$$

and hence

$$\frac{1}{1\;-\;H_{ii}}=\frac{n}{n\;-\;1\;-\;x_{i}^{2}}.$$

To identify when this covariance becomes positive, we make the approximation that $H_{ii}$ is constant, which implies $x_{i}^{2}=1$. This is not exact, but for small correlations between x and y, this introduces minimal error. With this approximation,

$$Cov(y,{\hat{\eta }}^{loo})\approx 1-\frac{n}{n\;-\;2}+Cov{\left(y,x\right)}^{2}\cdot \frac{n}{n\;-\;2}.$$

This becomes positive when

$$Cov{\left(y,x\right)}^{2}>\frac{2}{n}.$$

The critical value for a significance test for a correlation using the t-distribution is:

$$r=\frac{t}{\sqrt{n\;-\;2\;+\;t^{2}}}$$

We achieve this when $t=\sqrt{2}$, which corresponds to a two-sided p-value of 0.16 for large n.

### Appendix C. Proof of Theorems in Section 7

We first prove Theorem 1. We will need the following result (see, e.g., (n.d.-p)).

#### Lemma 1.

*(Bernstein's inequality). Let* $Z_{1},\;\ldots \;,Z_{n}$ *be independent and identically distributed random variables such that* $E\left(Z_{1}\right)=0$ *and* $E\left(e^{\beta \left|Z_{1}\right|}\right)\leq 2$ *for some* β>0. *Then for all* t≥0,

$$P(|\frac{1}{n}\sum_{i=1}^{n}Z_{i}|\geq t)\leq 2\exp\left(-min\left\{\frac{n\;\beta^{2}t^{2}}{4},\frac{n\;\beta t}{2}\right\}\right).$$

Define random variables

$$A\;:=\frac{1}{n}\sum_{i=1}^{n}Y_{i}^{2},\;B_{j}:=\frac{1}{n}\sum_{i=1}^{n}X_{i,j}^{2},\;D_{j}:=\frac{1}{n}\sum_{i=1}^{n}Y_{i}X_{i,j}.$$

As a corollary of Bernstein's inequality, we obtain the following.

#### Corollary 1.

*There is a positive constant* $C_{3}$ *depending only on* $C_{1}$ *and* $C_{2}$ *such that for all* t≥0,

$$P(|A-E(A)|\geq t)\leq 2e^{-C_{3}nmin\left\{t^{2},\;t\right\}}$$

*ad the same bound holds for* $P\left(\left|B_{j}-E\left(B_{j}\right)\right|\geq t\right)$ *and* $P\left(\left|D_{j}-E\left(D_{j}\right)\right|\geq t\right)$ *for all* j.

##### Proof.

Let $Z_{i}:=Y_{i}^{2}-E(A)$. Then $E\left(Z_{i}\right)=0$, and for any t≥0,

$$P\left(\left|Z_{1}\right|\geq t\right)\leq P\left(Y_{1}^{2}\geq t\right)\leq C_{1}e^{-C_{2}t}.$$

Thus, for any $\beta \in \left(0,C_{2}\right)$,

$$\begin{array}{ll} E\left(e^{\beta \left|Z_{1}\right|}\right) & =\int_{0}^{\infty }\beta \;e^{\beta t}P\left(\left|Z_{1}\right|\geq t\right)\;dt\\ \; & \leq \int_{0}^{\infty }\beta \;e^{\beta t}C_{1}e^{-C_{2}t}dt\\ \; & =\frac{C_{1}\beta }{C_{2}\;-\;\beta }. \end{array}$$

Choosing β sufficiently small makes the upper bound ≤ 2, allowing us to apply Bernstein's inequality. A similar argument works for $B_{j}$. For $D_{j}$, take $Z_{i}:=Y_{i}X_{i,j}-E\left(D_{j}\right)$. Then note that $E\left(Z_{i}\right)=0$, and for any $t\geq 2\left|E\left(D_{j}\right)\right|$,

$$\begin{array}{ll} P\left(\left|Z_{1}\right|\geq t\right) & \leq P\left(\left|Y_{1}X_{1,j}\right|\geq t/2\right)\\ \; & \leq P\left(Y_{1}^{2}\geq t/2\right)+P\left(X_{1,j}^{2}\geq t/2\right)\\ \; & \leq 2C_{1}e^{-C_{2}t/2}. \end{array}$$

Since $\left|E\left(D_{j}\right)\right|$ can be bounded above by a number that depends only on $C_{1}$ and $C_{2}$, this shows that there are constants $C_{3}$ and $C_{4}$ depending only on $C_{1}$ and $C_{2}$ such that for all t≥0,

$$P\left(\left|Z_{1}\right|\geq t\right)\leq C_{3}e^{-C_{4}t}.$$

The proof is now completed by proceeding as before.

■

We also obtain the following second corollary.

#### Corollary 2.

*There are positive constants* $C_{5},\;C_{6}$ *and* $C_{7}$ *depending only on* $C_{0},\;C_{1}$ *and* $C_{2}$ *such that for any* $t\in \left[0,C_{5}\right]$ *and any* i *and* $j,\;P\left(\left|{\hat{\beta }}_{j}^{-i}-\beta_{j}\right|\geq t\right)$ *and* $P\left(\left|{\hat{\alpha }}_{j}^{-i}-\alpha_{j}\right|\geq t\right)$ *are bounded above by* $C_{6}e^{-C_{7}nt^{2}}$.

##### Proof.

Fix i,j. Let

$$\begin{array}{l} Q_{1}:=\frac{1}{n\;-\;1}\sum_{k\neq i}Y_{k}X_{k,j},\;Q_{2}:=\frac{1}{n\;-\;1}\sum_{k\neq i}Y_{k},\\ Q_{3}:=\frac{1}{n\;-\;1}\sum_{k\neq i}X_{k,j},\;Q_{4}:=\frac{1}{n\;-\;1}\sum_{k\neq i}X_{k,j}^{2}. \end{array}$$

Using the same approach as in Corollary 1 and the fact that n-1≥n/2 (because n≥2), we deduce the concentration inequality

(B.1)
$$P\left(\left|Q_{l}-E\left(Q_{1}\right)\right|\geq t\right)\leq 2e^{-Knmin\left\{t^{2},\;t\right\}}$$

for each 1≤l≤4. Now, note that $E\left(Q_{4}\right)=E\left(X_{j}^{2}\right)$ and $E\left(Q_{3}\right)=E\left(X_{j}\right)$. Moreover, recall that $var\left(X_{j}\right)=E\left(X_{j}^{2}\right)-{\left(E\left(X_{j}\right)\right)}^{2}\geq C_{0}>0$. Combining these observations with the above inequality, we see that there are positive constants $K_{1}$ and $K_{2}$ such that

(B.2)
$$P(|Q_{4}-Q_{3}^{2}|<C_{0}/2)\leq K_{1}e^{-K_{2}n}.$$

Since

$${\hat{\beta }}_{j}^{-i}=\frac{Q_{1}\;-\;Q_{2}Q_{3}}{Q_{4}\;-\;Q_{3}^{2}},$$

the inequality (B.2) gives

(B.3)
$$\begin{array}{ll} P\left(\left|{\hat{\beta }}_{j}^{-i}-\beta_{j}\right|\geq t\right) & =P\left(\left|\frac{Q_{1}\;-\;Q_{2}Q_{3}\;-\;\left(Q_{4}\;-\;Q_{3}^{2}\right)\beta_{j}}{Q_{4}\;-\;Q_{3}^{2}}\right|\geq t\right)\\ \; & \leq P\left(\left|Q_{1}-Q_{2}Q_{3}-\left(Q_{4}-Q_{3}^{2}\right)\beta_{j}\right|\geq \frac{C_{0}t}{2}\right)+K_{1}e^{-K_{2}n}. \end{array}$$

Now take any s>0, and suppose that $\left|Q_{l}-E\left(Q_{l}\right)\right|<s$ for 1≤l≤4. Then we also have

$$\left|Q_{3}^{2}-{\left(E\left(Q_{3}\right)\right)}^{2}\right|\leq {\left|Q_{3}-E\left(Q_{3}\right)\right|}^{2}+2\left|E\left(Q_{3}\right)\right|\left|Q_{3}-E\left(Q_{3}\right)\right|<s^{2}+K_{3}s,$$

where $K_{3}$ depends only on $C_{1}$ and $C_{2}$. Similarly,

$$\left|Q_{2}Q_{3}-E\left(Q_{2}\right)E\left(Q_{3}\right)\right|<K_{4}s,$$

where $K_{4}$ depends only on $C_{1}$ and $C_{2}$. Thus, we have

$$\begin{array}{l} \left|\left(Q_{1}-Q_{2}Q_{3}-\left(Q_{4}-Q_{3}^{2}\right)\beta_{j}\right)-\left(E\left(Q_{1}\right)-E\left(Q_{2}\right)E\left(Q_{3}\right)-\left(E\left(Q_{4}\right)-{\left(E\left(Q_{3}\right)\right)}^{2}\right)\beta_{j}\right)\right|\\ <K_{5}s+K_{6}s^{2}, \end{array}$$

where $K_{5}$ and $K_{6}$ depend only on $C_{0},\;C_{1}$ and $C_{2}$. But

$$\begin{array}{l} E\left(Q_{1}\right)-E\left(Q_{2}\right)E\left(Q_{3}\right)-\left(E\left(Q_{4}\right)-{\left(E\left(Q_{3}\right)\right)}^{2}\right)\beta_{j}\\ =E\left(YX_{j}\right)-E(Y)E\left(X_{j}\right)-\left(E\left(X_{j}^{2}\right)-{\left(E\left(X_{j}\right)\right)}^{2}\right)\frac{E\left(YX_{j}\right)\;-\;E(Y)E\left(X_{j}\right)}{E\left(X_{j}^{2}\right)\;-\;{\left(E\left(X_{j}\right)\right)}^{2}}=0. \end{array}$$

Plugging this into the previous display shows that

(B.4)
$$P\left(\left|Q_{1}-Q_{2}Q_{3}-\left(Q_{4}-Q_{3}^{2}\right)\beta_{j}\right|\geq K_{5}s+K_{6}s^{2}\right)\leq \sum_{l=1}^{4}P\left(\left|Q_{l}-E\left(Q_{1}\right)\right|\geq s\right).$$

Choosing s to solve $K_{5}s+K_{6}s^{2}=C_{0}t/2$, and combining equations (B.1), (B.3), and (B.4) yields the proof of the claimed concentration inequality for ${\hat{\beta }}_{j}^{-i}$. The proof for ${\hat{\alpha }}_{j}^{-i}$ is similar. We omit the details.

■

We are now ready to prove Theorem 1.

##### Proof of Theorem 1.

Throughout this proof, $K_{1},K_{2},\;\ldots \;$ will denote positive constants that may depend only on $C_{0},\;C_{1},\;C_{2},\;\theta$ and |S| and nothing else, whose values may change from line to line. Also, we will denote by o(1) any random variable Z such that

$$P(|Z|\geq t)\leq K_{1}ne^{-K_{2}nt^{2}}$$

for all $t\in \left[0,K_{3}\right]$ (for some $K_{1},\;K_{2},\;K_{3}$ according to the above convention), and by O(1) any random variable Z such that $\left|Z-K_{1}\right|=o(1)$ for some $K_{1}$. Define

$$\tilde{L}\left(\theta_{0},\theta_{1},\;\ldots \;,\theta_{p}\right)\;:=\frac{1}{n}\sum_{i=1}^{n}{\left(Y_{i}-\theta_{0}-\theta_{1}\left({\hat{Y}}_{i,1}-{\hat{\alpha }}_{1}\right)-\cdots -\theta_{p}\left({\hat{Y}}_{i,p}-{\hat{\alpha }}_{p}\right)\right)}^{2}+\lambda \sum_{j=1}^{p}\theta_{j}.$$

Note that $\left({{\hat{\theta }}_{0}}^{\prime },{\hat{\theta }}_{1},\;\ldots \;,{\hat{\theta }}_{p}\right)$ minimizes $\tilde{L}$ under ${\hat{\theta }}_{j}\geq 0$ for all 1≤j≤p if and only if $\left({\hat{\theta }}_{0},{\hat{\theta }}_{1},\;\ldots \;,{\hat{\theta }}_{p}\right)$ minimizes L under the same constraint, where

$${\hat{\theta }}_{0}={{\hat{\theta }}_{0}}^{\prime }-\sum_{j=1}^{p}{\hat{\theta }}_{j}{\hat{\alpha }}_{j}.$$

Note that this means ${\hat{\gamma }}_{0}={{\hat{\theta }}_{0}}^{\prime }$. We will henceforth consider this modified optimization problem. Note that for each 1≤j≤p, and for any $\theta_{0}\in R$ and $\theta_{1},\;\ldots \;,\theta_{p}\geq 0$,

(B.5)
$$\begin{array}{ll} {\tilde{L}}_{j} & :=\frac{\partial \tilde{L}}{\partial \theta_{j}}\\ \; & =-\frac{2}{n}\sum_{i=1}^{n}\left(Y_{i}-\theta_{0}-\theta_{1}\left({\hat{Y}}_{i,1}-{\hat{\alpha }}_{1}\right)-\cdots -\theta_{p}\left({\hat{Y}}_{i,p}-{\hat{\alpha }}_{p}\right)\right)\left({\hat{Y}}_{i,j}-{\hat{\alpha }}_{j}\right)+\lambda . \end{array}$$

By an application of the Cauchy-Schwarz inequality, we get

$$\begin{array}{l} \left|\frac{1}{n}\sum_{i=1}^{n}\left(Y_{i}-\theta_{0}-\theta_{1}\left({\hat{Y}}_{i,1}-{\hat{\alpha }}_{1}\right)-\cdots -\theta_{p}\left({\hat{Y}}_{i,p}-{\hat{\alpha }}_{p}\right)\right)\left({\hat{Y}}_{i,j}-{\hat{\alpha }}_{j}\right)\right|\\ \leq {\left(\frac{1}{n}\sum_{i=1}^{n}{\left(Y_{i}-\theta_{0}-\theta_{1}\left({\hat{Y}}_{i,1}-{\hat{\alpha }}_{1}\right)-\cdots -\theta_{p}\left({\hat{Y}}_{i,p}-{\hat{\alpha }}_{p}\right)\right)}^{2}\right)}^{1/2}{\left(\frac{1}{n}\sum_{i=1}^{n}{\left({\hat{Y}}_{i,j}-{\hat{\alpha }}_{j}\right)}^{2}\right)}^{1/2}\\ \leq \sqrt{\tilde{L}\left(\theta_{0},\;\ldots \;,\theta_{p}\right)}{\left(\frac{1}{n}\sum_{i=1}^{n}{\left({\hat{Y}}_{i,j}-{\hat{\alpha }}_{j}\right)}^{2}\right)}^{1/2}. \end{array}$$

Now, note that

$$\begin{array}{ll} \frac{1}{n}\sum_{i=1}^{n}{\left({\hat{Y}}_{i,j}-{\hat{\alpha }}_{j}\right)}^{2} & \leq \frac{2}{n}\sum_{i=1}^{n}{\left({\hat{Y}}_{i,j}-{\hat{\alpha }}_{j}^{-i}\right)}^{2}+\frac{2}{n}\sum_{i=1}^{n}{\left({\hat{\alpha }}_{j}^{-i}-{\hat{\alpha }}_{j}\right)}^{2}\\ \; & \leq \frac{2}{n}\sum_{i=1}^{n}{\hat{\beta }}_{i,j}^{2}X_{i,j}^{2}+\frac{4}{n}\sum_{i=1}^{n}{\left({\hat{\alpha }}_{j}^{-i}-\alpha_{j}\right)}^{2}+4{\left({\hat{\alpha }}_{j}-\alpha_{j}\right)}^{2}\\ \; & \leq \left(\frac{2}{n}\sum_{i=1}^{n}X_{i,j}^{2}\right)\underset{1\leq i\leq n}{max}{\left({\hat{\beta }}_{j}^{-i}\right)}^{2}+4max\left\{{\left({\hat{\alpha }}_{j}-\alpha_{j}\right)}^{2},\underset{1\leq i\leq n}{max}{\left({\hat{\alpha }}_{j}^{-i}-\alpha_{j}\right)}^{2}\right\}. \end{array}$$

Let $M_{j}^{\beta }$ and $M_{j}^{\alpha }$ denote the two maxima on the right. Combining the previous three displays, we get that for $\theta_{0}\in R$ and $\theta_{1},\;\ldots \;,\theta_{p}\geq 0$,

(B.6)
$${\tilde{L}}_{j}\left(\theta_{0},\;\ldots \;,\theta_{p}\right)\geq \lambda -\sqrt{\tilde{L}\left(\theta_{0},\;\ldots \;,\theta_{p}\right)\left(2B_{j}M_{j}^{\beta }+4M_{j}^{\alpha }\right)}.$$

Take any j∈{1,…,p}\S. Suppose that ${\hat{\gamma }}_{j}\neq 0$. Then ${\hat{\theta }}_{j}>0$. This implies that

$${\tilde{L}}_{j}\left({\hat{\gamma }}_{0},{\hat{\theta }}_{1},\;\ldots \;,{\hat{\theta }}_{p}\right)=0,$$

because otherwise, we can slightly perturb ${\hat{\theta }}_{j}$ while maintaining the nonnegativity constraint and decreasing the value of L. By inequality (B.6), this implies that if ${\hat{\theta }}_{j}>0$, then we must have

$$\tilde{L}\left({\hat{\gamma }}_{0},{\hat{\theta }}_{1},\;\ldots \;,{\hat{\theta }}_{p}\right)\left(2B_{j}M_{j}^{\beta }+4M_{j}^{\alpha }\right)\geq \lambda^{2}.$$

But note that

$$\tilde{L}\left({\hat{Y}}_{0},{\hat{\theta }}_{1},\;\ldots \;,{\hat{\theta }}_{p}\right)\leq \tilde{L}(0,0,\;\ldots \;,0)=A.$$

Thus, we conclude that if ${\hat{\theta }}_{j}>0$, then

$$\lambda^{2}\leq A\left(2B_{j}M_{j}^{\beta }+4M_{j}^{\alpha }\right).$$

Thus,

$$\begin{array}{ll} P\left({\hat{\theta }}_{j}>0\right)\leq & P(A>2\;E(A))+P\left(B_{j}>2\;E\left(B_{j}\right)\right)\\ \; & +P\left(M_{j}^{\beta }\geq \frac{\lambda^{2}}{16\;E(A)E\left({\;B}_{j}\right)}\right)+P\left(M_{j}^{\alpha }\geq \frac{\lambda^{2}}{16\;E(A)}\right). \end{array}$$

By Corollary 1, the first two probabilities on the right are bounded above by $K_{1}e^{-K_{2}n}$. Next, note that if

(B.7)
$$K_{3}\left|\beta_{j}\right|\leq \lambda \leq K_{4},$$

for some sufficiently small $K_{3}$ and $K_{4}$, then by Corollary 2,

$$\begin{array}{ll} P\left(M_{j}^{\beta }\geq \frac{\lambda^{2}}{16\;E(A)\;E\left(B_{j}\right)}\right) & \leq n\;P\left({\hat{\beta }}_{1,j}^{2}\geq \frac{\lambda^{2}}{16\;E(A)\;E\left(B_{j}\right)}\right)\\ \; & \leq n\;P\left(\left|{\hat{\beta }}_{1,j}-\beta_{j}\right|\geq K_{5}\lambda \right)\leq K_{6}ne^{-K_{7}n\lambda^{2}}. \end{array}$$

Similarly, the same bound holds for the tail probability of $M_{j}^{\alpha }$. Combining, we get that under the condition (B.7),

$$P\left({\hat{\theta }}_{j}>0\right)\leq K_{8}ne^{-K_{9}n\lambda^{2}},$$

and thus,

(B.8)
$$P\left({\hat{\gamma }}_{j}\neq 0\;\text{for}\;\text{some}\;j\notin S\cup \{0\}\right)\leq P\left(E^{c}\right)\leq K_{8}pne^{-K_{9}n\lambda^{2}},$$

where E denotes the event that ${\hat{\theta }}_{j}=0$ for all j∉S∪{0}. Suppose that E happens. Take any k∈S. If ${\hat{\theta }}_{k}\neq 0$, then ${\tilde{L}}_{k}\left({\hat{Y}}_{0},{\hat{\theta }}_{1},\;\ldots \;,{\hat{\theta }}_{p}\right)=0$, whereas if ${\hat{\theta }}_{k}=0$, then ${\tilde{L}}_{k}\left({\hat{Y}}_{0},{\hat{\theta }}_{1},\;\ldots \;,{\hat{\theta }}_{p}\right)\geq 0$ and $\left(\gamma_{k}-{\hat{\gamma }}_{k}\right)/\beta_{k}=\gamma_{k}/\beta_{k}>0$. Thus, in either case, we have

$$\frac{\gamma_{k}\;-\;{\hat{\gamma }}_{k}}{\beta_{k}}{\tilde{L}}_{k}\left({\hat{\gamma }}_{0},{\hat{\theta }}_{1},\;\ldots \;,{\hat{\theta }}_{p}\right)\geq 0.$$

By the formula (B.5) for ${\tilde{L}}_{j}$, this shows that

(B.9)
$$\begin{array}{ll} \frac{\lambda \left(\gamma_{k}\;-\;{\hat{\gamma }}_{k}\right)}{2\beta_{k}} & \geq \left(\frac{\gamma_{k}\;-\;{\hat{\gamma }}_{k}}{\beta_{k}}\right)\frac{1}{n}\sum_{i=1}^{n}\left(Y_{i}-{\hat{\gamma }}_{0}-\sum_{j\in S}{\hat{\theta }}_{j}\left({\hat{Y}}_{i,j}-{\hat{\alpha }}_{j}\right)\right)\left({\hat{Y}}_{i,k}-{\hat{\alpha }}_{k}\right)\\ \; & =\frac{\gamma_{k}\;-\;{\hat{\gamma }}_{k}}{n\beta_{k}}\sum_{i=1}^{n}\left[\gamma_{0}-{\hat{\gamma }}_{0}+\sum_{j\in S}\left(\gamma_{j}X_{i,j}-{\hat{\theta }}_{j}\left({\hat{Y}}_{i,j}-{\hat{\alpha }}_{j}\right)\right)\right]\left({\hat{Y}}_{i,k}-{\hat{\alpha }}_{k}\right)\\ \; & \;+\frac{\gamma_{k}\;-\;{\hat{\gamma }}_{k}}{n\beta_{k}}\sum_{i=1}^{n}\epsilon_{i}\left({\hat{Y}}_{i,k}-{\hat{\alpha }}_{k}\right). \end{array}$$

Define

$$M\;:=\underset{1\leq i\leq n,j\in S}{max}\left(\left|{\hat{\beta }}_{j}^{-i}-\beta_{j}\right|+\left|{\hat{\beta }}_{j}-\beta_{j}\right|\right).$$

Then

(B.10)
$$\left|\left({\hat{Y}}_{i,k}-{\hat{\alpha }}_{k}\right)-\beta_{k}X_{i,k}\right|=\left|{\hat{\beta }}_{i,k}-\beta_{k}\right|\left|X_{i,k}\right|\leq M\left|X_{i,k}\right|,$$

and since ${\hat{\gamma }}_{j}={\hat{\theta }}_{j}{\hat{\beta }}_{j}$,

(B.11)
$$\begin{array}{ll} \left|{\hat{\theta }}_{j}\left({\hat{Y}}_{i,j}-{\hat{\alpha }}_{j}\right)-{\hat{\gamma }}_{j}X_{i,j}\right| & =\left|{\hat{\theta }}_{j}{\hat{\beta }}_{j}^{-i}X_{i,j}-{\hat{\theta }}_{j}{\hat{\beta }}_{j}X_{i,j}\right|\\ \; & =\left|{\hat{\beta }}_{j}^{-i}-{\hat{\beta }}_{j}\right|\left|{\hat{\theta }}_{j}X_{i,j}\right|\leq M\left|{\hat{\theta }}_{j}\right|\left|X_{i,j}\right|. \end{array}$$

By (B.10) and (B.11), we have

(B.12)
$$\begin{array}{l} \left|{\hat{\theta }}_{j}\left({\hat{Y}}_{i,j}-{\hat{\alpha }}_{j}\right)\left({\hat{Y}}_{i,k}-{\hat{\alpha }}_{k}\right)-{\hat{\gamma }}_{j}\beta_{k}X_{i,j}X_{i,k}\right|\\ \leq \left|{\hat{\theta }}_{j}\left({\hat{Y}}_{i,j}-{\hat{\alpha }}_{j}\right)-{\hat{\gamma }}_{j}X_{i,j}\right|\left|{\hat{Y}}_{i,k}-{\hat{\alpha }}_{k}\right|+\left|{\hat{\gamma }}_{j}X_{i,j}\right|\left|\left({\hat{Y}}_{i,k}-{\hat{\alpha }}_{k}\right)-\beta_{k}X_{i,k}\right|\\ \leq M\left|{\hat{\theta }}_{j}\right|\left|X_{i,j}\right|\left(\left|\beta_{k}X_{i,k}\right|+M\left|X_{i,k}\right|\right)+M\left|{\hat{\gamma }}_{j}X_{i,k}X_{i,j}\right| \end{array}$$

Let $S^{\prime }\;:=S\cup \{0\}$. Define $X_{0}\equiv 1$ and $X_{i,0}\equiv 1$ for 1≤i≤n. Combining equations (B.9), (B.10), (B.11), and (B.12), we get

(B.13)
$$\begin{array}{l} \frac{\lambda \left(\gamma_{k}\;-\;{\hat{\gamma }}_{k}\right)}{2\beta_{k}}-\left[\left(\gamma_{k}-{\hat{\gamma }}_{k}\right)\sum_{j\in S\prime }\left(\gamma_{j}-{\hat{\gamma }}_{j}\right)\left(\frac{1}{n}\sum_{i=1}^{n}X_{i,j}X_{i,k}\right)+\left(\gamma_{k}-{\hat{\gamma }}_{k}\right)\frac{1}{n}\sum_{i=1}^{n}\epsilon_{i}X_{i,k}\right]\\ \geq -K_{5}\left(M+M^{2}\right)\left|\frac{\gamma_{k}\;-\;{\hat{\gamma }}_{k}}{n\beta_{k}}\right|\sum_{i=1}^{n}(\left|\gamma_{0}-{\hat{\gamma }}_{0}\right|\left|X_{i,k}\right|\\ \;+\sum_{j\in S}\left(1+\left|{\hat{\theta }}_{j}\right|+\left|{\hat{\gamma }}_{j}\right|\right)\left(\left|X_{i,j}\right|+\left|\epsilon_{i}\right|\right)\left|X_{i,k}\right|). \end{array}$$

Next note that ${\tilde{L}}_{0}\left({\hat{\gamma }}_{0},{\hat{\theta }}_{1},\;\ldots \;,{\hat{\theta }}_{p}\right)=0$, where ${\tilde{L}}_{0}$ denotes the derivative of $\tilde{L}$ is the zeroth coordinate. This means that

$$\begin{array}{ll} 0 & =\frac{1}{n}\sum_{i=1}^{n}\left(Y_{i}-{\hat{\gamma }}_{0}-\sum_{j\in S}{\hat{\theta }}_{j}\left({\hat{Y}}_{i,j}-{\hat{\alpha }}_{j}\right)\right)\\ \; & =\frac{1}{n}\sum_{i=1}^{n}\left[\gamma_{0}-{\hat{\gamma }}_{0}+\sum_{j\in S}\left(\gamma_{j}X_{i,j}-{\hat{\theta }}_{j}\left({\hat{Y}}_{i,j}-{\hat{\alpha }}_{j}\right)\right)\right]+\frac{1}{n}\sum_{i=1}^{n}\epsilon_{i}. \end{array}$$

Proceeding as before (and recalling that $X_{i,0}=1$), this gives

(B.14)
$$\begin{array}{l} \left(\gamma_{0}-{\hat{\gamma }}_{0}\right)\sum_{j\in S^{\prime }}\left(\gamma_{j}-{\hat{\gamma }}_{j}\right)\left(\frac{1}{n}\sum_{i=1}^{n}X_{i,j}X_{i,0}\right)+\left(\gamma_{0}-{\hat{\gamma }}_{0}\right)\frac{1}{n}\sum_{i=1}^{n}\epsilon_{i}X_{i,0}\\ \leq \frac{M\left|\gamma_{0}\;-\;{\hat{\gamma }}_{0}\right|}{n}\sum_{j\in S}\left|{\hat{\theta }}_{j}X_{i,j}\right|. \end{array}$$

Let $\Delta \;:=\underset{j\in S^{\prime }}{max}\left|{\hat{\gamma }}_{j}-\gamma_{j}\right|$. For each j∈S, let $\theta_{j}:=\gamma_{j}/\beta_{j}$. Recall the quantity $M_{2}$ from the statement of the theorem. Note that if

(B.15)
$$M<\frac{1}{2}M_{2},$$

we get that for each j∈S,

$$\begin{array}{ll} \left|{\hat{\theta }}_{j}-\theta_{j}\right| & =\left|\frac{{\hat{\gamma }}_{j}}{{\hat{\beta }}_{j}}-\frac{\gamma_{j}}{\beta_{j}}\right|\\ \; & \leq \frac{\left|{\hat{\gamma }}_{j}\;-\;\gamma_{j}\right|}{\left|{\hat{\beta }}_{j}\right|}+\left|\gamma_{j}\right|\left|\frac{1}{{\hat{\beta }}_{j}}-\frac{1}{\beta_{j}}\right|\\ \; & \leq K_{6}(\Delta +M). \end{array}$$

Combining this observation with the inequalities (B.13) and (B.14), and summing over $k\in S^{\prime }$, we get that

(B.16)
$$\sum_{j,k\in S\prime }{\hat{\sigma }}_{j,k}\left(\gamma_{j}-{\hat{\gamma }}_{j}\right)\left(\gamma_{k}-{\hat{\gamma }}_{k}\right)\leq K_{7}\lambda \Delta +K_{8}\Delta Q_{1}+K_{9}\left(M+M^{2}\right)\Delta \left(\Delta +M\right)Q_{2},$$

where

$${\hat{\sigma }}_{j,k}:=\frac{1}{n}\sum_{i=1}^{n}X_{i,j}X_{i,k},\;Q_{1}:=\underset{j\in S\prime }{max}\left|\frac{1}{n}\sum_{i=1}^{n}\epsilon_{i}X_{i,j}\right|,\;Q_{2}:=\underset{j\in S\prime }{max}\frac{1}{n}\sum_{i=1}^{n}X_{i,j}^{2}.$$

Let $\hat{\eta }$ be the smallest eigenvalue of the positive semidefinite matrix ${\left({\hat{\sigma }}_{j,k}\right)}_{j,k\in S\prime }$, so that

$$\sum_{j,k\in S\prime }{\hat{\sigma }}_{j,k}\left(\gamma_{j}-{\hat{\gamma }}_{j}\right)\left(\gamma_{k}-{\hat{\gamma }}_{k}\right)\geq \hat{\eta }\sum_{j\in S\prime }{\left({\hat{\gamma }}_{j}-\gamma_{j}\right)}^{2}\geq \hat{\eta }\Delta^{2}.$$

Combining this with (B.16) and rearranging, we get

$$\left(\hat{\eta }-K_{9}\left(M+M^{2}\right)Q_{2}\right)\Delta^{2}\leq \left(K_{7}\lambda +K_{8}Q_{1}+K_{9}\left(M+M^{2}\right)MQ_{2}\right)\Delta$$

If the coefficient of $\Delta^{2}$ on the left is positive, this gives

$$\Delta \leq \frac{K_{7}\lambda \;+\;K_{8}Q_{1}\;+\;K_{9}\left(M\;+\;M^{2}\right)MQ_{2}}{\hat{\eta }\;-\;K_{9}\left(M\;+\;M^{2}\right)Q_{2}}.$$

Now, using Bernstein's inequality, it is easy to show that $Q_{1}=o(1)$ and $Q_{2}=O(1)$ (following the conventions introduced at the beginning of the proof). By Corollary 2, we know that M=o(1). Again by Bernstein's inequality, ${\hat{\sigma }}_{j,k}=E\left(X_{j}X_{k}\right)+o(1)$ for each j,k∈S. From this, it is easy to deduce via standard matrix inequalities that $\hat{\eta }=\eta_{0}+o(1)$, where $\eta_{0}$ is the minimum eigenvalue of ${\left(E\left(X_{j}X_{k}\right)\right)}_{j,k\in S\prime }$. Recalling that $X_{0}=1$, we see that this is equal to the minimum eigenvalue η of the covariance matrix of ${\left(X_{j}\right)}_{j\in S}$. Combining all of these, it is now straightforward that under the condition (B.7),

$$P\left(E\cap \left\{\Delta >K_{10}\lambda \right\}\right)\leq K_{11}ne^{-K_{12}n\lambda^{2}}.$$

Together with (B.8), this completes the proof.

■

##### Proof of Theorem 2.

Note that since $Y=\gamma_{0}+\sum_{j\in S}\gamma_{j}X_{j}+\epsilon$, and ϵ is independent of the $X_{j}$'s, we have

$$\begin{array}{ll} \beta_{j} & =\frac{Cov\left(Y,\;X_{j}\right)}{Var\left(X_{j}\right)}\\ \; & =\frac{\sum_{k\in S}\gamma_{k}\;Cov\left(X_{k},\;X_{j}\right)}{Var\left(X_{j}\right)}\\ \; & =\gamma_{j}+\sum_{k\in S\backslash \{j\}}\gamma_{k}\frac{Cov\left(X_{k},\;X_{j}\right)}{Var\left(X_{j}\right)}=\gamma_{j}+\sum_{k\in S\backslash \{j\}}\gamma_{k}\delta_{k,j}. \end{array}$$

Since $\gamma_{j}\neq 0$, we may divide throughout by $\gamma_{j}$ and get

$$\frac{\beta_{j}}{\gamma_{j}}=1+\sum_{k\in S\backslash \{j\}}\frac{\gamma_{k}}{\gamma_{j}}\delta_{k,j}.$$

Now, by assumption, $\delta_{k,j}\geq 0$ whenever $\gamma_{k}/\gamma_{j}>0$, and $\delta_{k,j}\leq 0$ whenever $\gamma_{k}/\gamma_{j}<0$. Thus,

$$\sum_{k\in S\backslash \{j\}}\frac{\gamma_{k}}{\gamma_{j}}\delta_{k,j}\geq 0.$$

This shows that $\beta_{j}/\gamma_{j}\geq 1$. In particular, $\beta_{j}$ is nonzero and has the same sign as $\gamma_{j}$.

■

##### Proof of Theorem 3.

Note that

$$\begin{array}{ll} \beta_{j} & =\frac{Cov\left(Y,\;X_{j}\right)}{Var\left(X_{j}\right)}\\ \; & =\sum_{k\in S}\gamma_{k}\frac{Cov\left(X_{k},\;X_{j}\right)}{Var\left(X_{j}\right)}\\ \; & =\sum_{k\in S}\gamma_{k}\delta_{kj}. \end{array}$$

Thus,

$$\begin{array}{ll} \frac{\beta_{j}}{\gamma_{j}} & =\sum_{k\in S}\frac{\gamma_{k}}{\gamma_{j}}\delta_{kj}\\ \; & =\sum_{k\in A_{j}}\left|\frac{\gamma_{k}}{\gamma_{j}}\delta_{kj}\right|-\sum_{k\notin A_{j}}\left|\frac{\gamma_{k}}{\gamma_{j}}\delta_{kj}\right|\\ \; & =\frac{\sum_{k\in A_{j}}\left|\gamma_{k}\delta_{kj}\right|\;-\;\sum_{k\notin A_{j}}\left|\gamma_{k}\delta_{kj}\right|}{\left|\gamma_{j}\right|}. \end{array}$$

Since $\beta_{j}\gamma_{j}\geq 0$ if and only if $\beta_{j}/\gamma_{j}\geq 0$, this proves the claim.

■

### Appendix D. Proof of Theorem 4

Let $\gamma :=\left(\beta_{0},\beta \right)\in R^{p+1}$ and define ${\hat{\gamma }}^{OLS}$ and ${\hat{\gamma }}^{UR}$ similarly. Let X denote the n×(p+1) matrix whose columns are indexed from 0 to $p;\;X_{i,0}=1$ for each i, and for $1\leq j\leq p,\;X_{i,j}$ is as before. Let $Y\in R^{n}$ be the vector whose $i^{\text{th}}$ component is $Y_{i}$, and define ϵ similarly. Note that Y=Xγ+ϵ, and that ${\hat{\mu }}^{OLS}$ is the Euclidean projection on Y on the column space 𝒞 of X. Let

$$𝒟\;:=\left\{X\theta :\theta =\left(\theta_{0},\;\ldots \;,\theta_{p}\right)\in R^{p},\theta_{j}{\hat{\beta }}_{j}^{\mathrm{uni}}\geq 0\;\text{for}\;\text{all}\;1\leq j\leq p\right\},$$

so that 𝒟 is a random closed convex subset of 𝒞, and ${\hat{\mu }}^{UR}$ is the Euclidean projection of Y on 𝒟. Let E be the event that ${\hat{\beta }}_{j}^{\mathrm{uni}}\beta_{j}^{\mathrm{uni}}\geq 0$ for each 1≤j≤p. Note that if E happens, then μ∈𝒟.

#### Lemma 2.

*There is a positive constant* $C_{0}$ *depending only on* $\lambda_{0},\;\delta$, *and* σ *such that*

$$P(E)\geq 1-pe^{-C_{0}n}.$$

##### Proof.

If $\beta_{j}^{\mathrm{uni}}=0$, then ${\hat{\beta }}_{j}^{\mathrm{uni}}\beta_{j}^{\mathrm{uni}}\geq 0$ anyway. Thus,

$$\begin{array}{ll} P\left(E^{c}\right) & =P\left({\hat{\beta }}_{j}^{\mathrm{uni}}\beta_{j}^{\mathrm{uni}}<0\;\text{for}\;\text{some}\;j\;\text{such}\;\text{that}\;\beta_{j}^{\mathrm{uni}}\neq 0\right)\\ \; & \leq \sum_{j:{\;\beta }_{j}^{\mathrm{uni}}\neq 0}P\left({\hat{\beta }}_{j}^{\mathrm{uni}}\beta_{j}^{\mathrm{uni}}<0\right). \end{array}$$

Take any j such that $\beta_{j}^{\mathrm{uni}}\neq 0$. Then by standard results, conditional on X,

$${\hat{\beta }}_{j}^{\mathrm{uni}}∼N\left(\beta_{j}^{\mathrm{uni}},\frac{\sigma^{2}}{\sum_{i=1}^{n}{\left(X_{i,j}\;-\;{\bar{X}}_{j}\right)}^{2}}\right),$$

where ${\bar{X}}_{j}:=\frac{1}{n}\sum_{i=1}^{n}X_{i,j}$. Recall that $\left|\beta_{j}^{\mathrm{uni}}\right|\geq \delta$ and $Var\left(X_{i,j}\right)\geq \lambda_{0}$. From this, it is easy to deduce that there is a positive constant $C_{0}$ depending only on $\lambda_{0},\;\delta$, and σ such that $P\left(E^{c}\right)\leq pe^{-C_{0}n}$. This completes the proof.

■

#### Lemma 3.

If the event E happens, then

$${\left\|\mu -{\hat{\mu }}^{UR}\right\|}^{2}\leq {\left\|\mu -{\hat{\mu }}^{OLS}\right\|}^{2}-{\left\|{\hat{\mu }}^{OLS}-{\hat{\mu }}^{UR}\right\|}^{2}.$$

##### Proof.

Since ${\hat{\mu }}^{\text{OLS}}$ is the projection of Y on 𝒞 and 𝒞 is a subspace, it follows that $Y-{\hat{\mu }}^{\text{OLS}}$ is orthogonal to $x-{\hat{\mu }}^{OLS}$ for all x∈𝒞. Since 𝒟⊆𝒞, this implies that for any v∈𝒟,

$${\left\|Y-v\right\|}^{2}={\left\|Y-{\hat{\mu }}^{OLS}\right\|}^{2}+{\left\|{\hat{\mu }}^{OLS}-v\right\|}^{2}.$$

This shows that the projection of Y on 𝒟 (that is, ${\hat{\mu }}^{UR}$) is the same as the projection of ${\hat{\mu }}^{OLS}$ on 𝒟. Since 𝒟 is convex and μ∈𝒟 (because E has happened), $t\mu +(1-t){\hat{\mu }}^{UR}\in 𝒟$ for all t∈[0,1]. But then, by the preceding sentence,

$${\left\|{\hat{\mu }}^{OLS}-(t\mu +(1-t){\hat{\mu }}^{UR})\right\|}^{2}\geq {\left\|{\hat{\mu }}^{OLS}-{\hat{\mu }}^{UR}\right\|}^{2}$$

for all t∈[0,1]. In particular, the derivative of the left side with respect to t should be nonnegative at t=0. This shows that

$${\left({\hat{\mu }}^{OLS}-{\hat{\mu }}^{UR}\right)}^{T}({\hat{\mu }}^{UR}-\mu )\geq 0.$$

From this, we get

$$\begin{array}{ll} {\left\|{\hat{\mu }}^{OLS}-\mu \right\|}^{2}-{\left\|{\hat{\mu }}^{UR}-\mu \right\|}^{2} & ={\left\|{\hat{\mu }}^{OLS}-{\hat{\mu }}^{UR}\right\|}^{2}+2{\left({\hat{\mu }}^{OLS}-{\hat{\mu }}^{UR}\right)}^{T}({\hat{\mu }}^{UR}-\mu )\\ \; & \geq {\left\|{\hat{\mu }}^{OLS}-{\hat{\mu }}^{UR}\right\|}^{2}. \end{array}$$

This completes the proof.

■

#### Lemma 4.

*Let* Z *be an* n×p *matrix of independent and identically distributed* N(0,1) *random variables. Let* $1\in R^{n}$ *be the vector of all 1's, and let* $V\;:=Z-\frac{1}{n}11^{T}Z$. *Let* μ *be the minimum eigenvalue of* $\frac{1}{n}V^{T}V$ *and* v *be the maximum eigenvalue of* $\frac{1}{n}Z^{T}Z$. *There are positive constants* $C_{1},\;C_{2},\;\mu_{0},\;v_{0}$ *depending only on* $r_{0}$ *such that*

$$P\left(\mu \geq \mu_{0},v\leq v_{0}\right)\geq 1-C_{1}e^{-C_{2}n}.$$

##### Proof.

By basic facts from statistics, we know that $Z^{T}Z$ follows the Wishart distribution $W_{p}\left(n,I_{p}\right)$, and $V^{T}V∼W_{p}\left(n-1,I_{p}\right)$. The claimed bounds now follow from known results in the literature, such as (n.d.-q).

■

#### Lemma 5.

*Let* ${\hat{\lambda }}_{0}$ *and* ${\hat{\lambda }}_{1}$ *be the minimum and maximum eigenvalues of* $\frac{1}{n}X^{T}X$. *There are positive constants* $C_{3},\;C_{4},\;{\lambda_{0}}^{\prime },\;{\lambda_{1}}^{\prime }$ *depending only on* $\lambda_{0},\;\lambda_{1}$, *and* $r_{0}$ *such that*

$$P\left(\lambda_{0}^{\prime }\leq {\hat{\lambda }}_{0}\leq {\hat{\lambda }}_{1}\leq \lambda_{1}^{\prime }\right)\geq 1-C_{3}e^{-C_{4}n}.$$

##### Proof.

Let us write $X=\left[\begin{array}{ll} 1 & \tilde{X} \end{array}\right]$, where $\tilde{X}$ consists of columns 1,…,p of X and 1 denotes the first column, which has all 1's. Then for any $y=\left(y_{0},\tilde{y}\right)\in R^{p+1}$, where $y_{0}$ denotes the first coordinate of y, we have

(C.1)
$${\left\|\;Xy\;\right\|}^{2}={\left\|\;y_{0}1+\tilde{X}\tilde{Y}\;\right\|}^{2}.$$

Let $\Sigma^{1/2}$ denote the positive definite square-root of Σ. Then we can write $\tilde{X}=Z\Sigma^{1/2}$, where Z is an n×p matrix with independent and identically distributed N(0,1) random variables. Let $V\;:=Z-\frac{1}{n}11^{T}Z$. Let μ be the smallest eigenvalue of $\frac{1}{n}V^{T}V$ and v be the largest eigenvalue of $\frac{1}{n}Z^{T}Z$. By the above identity and the inequality ${\left\|\;u+v\;\right\|}^{2}\leq 2{{\left\|\;u\;\right\|}^{2}+2\|\;v\;\|}^{2}$, we get

$$\begin{array}{ll} \|\;Xy{\;\|}^{2} & ={\left\|\;y_{0}1+Z\Sigma^{1/2}\tilde{y}\;\right\|}^{2}\\ \; & \leq 2\;{\left\|\;y_{0}1\;\right\|}^{2}+2\;{\left\|\;Z\Sigma^{1/2}\tilde{y}\;\right\|}^{2}\\ \; & \leq 2y_{0}^{2}n+2nv{\left\|\;\Sigma^{1/2}\tilde{y}\;\right\|}^{2}\\ \; & \leq 2y_{0}^{2}n+2nv\lambda_{1}\|\;\tilde{y}{\;\|}^{2}\leq 2n\left(1+\lambda_{1}v\right)\|\;y{\;\|}^{2}. \end{array}$$

This shows that

(C.2)
$${\hat{\lambda }}_{1}\leq 2\left(1+\lambda_{1}v\right).$$

Next, let z be the projection of $Z\Sigma^{1/2}\tilde{y}$ on the span of 1, given by

$$z=n^{-1}11^{T}Z\Sigma^{1/2}\tilde{y}.$$

Then by (C.1),

(C.3)
$$\begin{array}{ll} \|\;Xy{\;\|}^{2} & ={\left\|\;y_{0}1-z\;\right\|}^{2}+\|\;\tilde{X}\tilde{y}-z{\;\|}^{2}\\ \; & \geq \|\;\tilde{X}\tilde{y}-z{\;\|}^{2}={\left\|\;Z\Sigma^{1/2}\tilde{y}-n^{-1}11^{T}Z\Sigma^{1/2}\tilde{y}\;\right\|}^{2}\\ \; & ={\left\|\;V\Sigma^{1/2}\tilde{y}\;\right\|}^{2}\geq n\mu {\left\|\;\Sigma^{1/2}\tilde{y}\;\right\|}^{2}\geq n\mu \lambda_{0}\;\|\;\tilde{y}{\;\|}^{2}. \end{array}$$

But by the first line of the above display, we also have

(C.4)
$$\|\;Xy{\;\|}^{2}\geq {\left\|\;y_{0}1-z\;\right\|}^{2}\geq {\left(\left\|\;y_{0}1\;\|-\|\;z\;\right\|\right)}^{2}={\left(\left|y_{0}\right|\sqrt{n}-\|\;z\;\|\right)}^{2}.$$

Now, note that

(C.5)
$$\|\;z\;\|=n^{-1}\left|1^{T}Z\Sigma^{1/2}\tilde{y}\right|\;\|\;1\;\|\leq n^{-1/2}\|\;1\;\|\|\;Z\Sigma^{1/2}\tilde{y}\;\|\leq \sqrt{n}v\lambda_{1}\|\;\tilde{y}\;\|.$$

So, if $v\lambda_{1}\|\;\tilde{y}\;\|\leq \frac{1}{2}\left|y_{0}\right|$, then by (C.4) and (C.5),

$$\|\;Xy{\;\|}^{2}\geq \frac{1}{4}y_{0}^{2}n=\left(\frac{1}{8}y_{0}^{2}+\frac{1}{8}y_{0}^{2}\right)n\geq \left(\frac{1}{8}y_{0}^{2}+\frac{4}{8}v^{2}\lambda_{1}^{2}\|\;\tilde{y}{\;\|}^{2}\right)n.$$

Without loss, let us assume that v and $\lambda_{1}$ were chosen so large that $4v^{2}\lambda_{1}^{2}\geq 1$. Then the above inequality gives

(C.6)
$$\|\;Xy{\;\|}^{2}\geq \left(\frac{1}{8}\left(\|\;y{\;\|}^{2}-\|\;\tilde{y}{\;\|}^{2}\right)+\frac{4}{8}v^{2}\lambda_{1}^{2}\|\;\tilde{y}{\;\|}^{2}\right)n\geq \frac{1}{8}\|\;y{\;\|}^{2}n.$$

On the other hand, if $v\lambda_{1}\|\;\tilde{y}\;\|>\frac{1}{2}\left|y_{0}\right|$, then by (C.3),

(C.7)
$$\begin{array}{ll} \|\;Xy{\;\|}^{2} & \geq n\mu \lambda_{0}\left(\frac{1}{2}\|\;\tilde{y}{\;\|}^{2}+\frac{1}{2}\|\;\tilde{y}{\;\|}^{2}\right)\\ \; & \geq n\mu \lambda_{0}\left(\frac{1}{2}\|\;\tilde{y}{\;\|}^{2}+\frac{1}{8v^{2}\lambda_{1}^{2}}y_{0}^{2}\right)\\ \; & =n\mu \lambda_{0}\left(\frac{1}{2}\|\;\tilde{y}{\;\|}^{2}+\frac{1}{8v^{2}\lambda_{1}^{2}}\left(\|\;y{\;\|}^{2}-\|\;\tilde{y}{\;\|}^{2}\right)\right)\geq \frac{n\mu \lambda_{0}}{8v^{2}\lambda_{1}^{2}}\|\;y{\;\|}^{2}, \end{array}$$

where the last inequality follows from the assumption that $4v^{2}\lambda_{1}^{2}\geq 1$. Combining the inequalities (C.6) and (C.7), we see that

(C.8)
$${\hat{\lambda }}_{0}\geq min\left\{\frac{1}{8},\frac{\mu \lambda_{0}}{8v^{2}\lambda_{1}^{2}}\right\}.$$

Inequalities (C.2) and (C.8) and Lemma 4 complete the proof.

■

We are now ready to prove Theorem 4. First, note that

$$\begin{array}{ll} {\left\|{\hat{\mu }}^{OLS}-{\hat{\mu }}^{UR}\right\|}^{2} & ={\left\|X{\hat{\gamma }}^{OLS}-X{\hat{\gamma }}^{UR}\right\|}^{2}\geq n{\hat{\lambda }}_{0}{\left\|{\hat{\gamma }}^{OLS}-{\hat{\gamma }}^{UR}\right\|}^{2}\\ \; & =n{\hat{\lambda }}_{0}{\left({\hat{\beta }}_{0}^{OLS}-{\hat{\alpha }}_{0}^{UR}\right)}^{2}+n{\hat{\lambda }}_{0}\sum_{j=1}^{p}{\left({\hat{\beta }}_{j}^{OLS}-{\hat{\alpha }}_{j}^{UR}\right)}^{2}. \end{array}$$

By the definition of ${\hat{\alpha }}^{UR},\;{\hat{\alpha }}_{j}^{UR}{\hat{\beta }}_{j}^{\mathrm{uni}}\geq 0$ for each j; that is, ${\hat{\alpha }}_{j}^{UR}$ and ${\hat{\beta }}_{j}^{\mathrm{uni}}$ are on the same side of zero on the real line (including the possibility that one or both may be equal to zero). Thus, if E happens and ${\hat{\beta }}_{j}^{\text{OLS}}\beta_{j}^{\mathrm{uni}}<0$, then ${\hat{\beta }}_{j}^{\text{OLS}}$ and ${\hat{\alpha }}_{j}^{\text{UR}}$ are on opposite sides of zero (again, including the possibility that one or both may be equal to zero), and hence,

$${\left({\hat{\beta }}_{j}^{OLS}-{\hat{\alpha }}_{j}^{UR}\right)}^{2}\geq {\left({\hat{\beta }}_{j}^{OLS}\right)}^{2}.$$

(Note that this does not hold if we weaken the condition to ${\hat{\beta }}_{j}^{\text{OLS}}\beta_{j}^{\mathrm{uni}}\leq 0$. This is because this weakening leaves open the possibility that $\beta_{j}^{\mathrm{uni}}=0$ and ${\hat{\beta }}_{j}^{\text{OLS}},\;{\hat{\alpha }}_{j}^{\text{UR}}$ are on the same side of zero.) Combining, we see that if E happens, then

(C.9)
$${\left\|{\hat{\mu }}^{OLS}-{\hat{\mu }}^{UR}\right\|}^{2}\geq n{\hat{\lambda }}_{0}\sum_{j\;:{\hat{\beta }}_{j}^{OLS}\beta_{j}^{uni}<0}{\left({\hat{\beta }}_{j}^{OLS}\right)}^{2}.$$

Take any j such that $\beta_{j}=0$ and $\beta_{j}^{\mathrm{uni}}\neq 0$. Conditional on X,

(C.10)
$${\hat{\beta }}_{j}^{OLS}∼N\left(0,\sigma^{2}\theta_{j}\right),$$

where $\theta_{0},\;\ldots \;,\theta_{p}$ are the diagonal elements of ${\left(X^{T}X\right)}^{-1}$. Since $\theta_{j}\geq {\left(n{\hat{\lambda }}_{1}\right)}^{-1}$, this shows that

(C.11)
$$E\left({\left({\hat{\beta }}_{j}^{OLS}\right)}^{2}1_{\left\{{\hat{\beta }}_{j}^{OLS}\beta_{j}^{uni}<0\right\}}|X\right)=\frac{1}{2}E\left({\left({\hat{\beta }}_{j}^{OLS}\right)}^{2}|X\right)\geq \frac{\sigma^{2}}{2n{\hat{\lambda }}_{1}}.$$

Let F be the event that ${\lambda_{0}}^{\prime }\leq {\hat{\lambda }}_{0}\leq {\hat{\lambda }}_{1}\leq {\lambda_{1}}^{\prime }$, where ${\lambda_{0}}^{\prime },\;{\lambda_{1}}^{\prime }$ are the constants from Lemma 5, and let H:=E∩F. Then by the inequality (C.9),

(C.12)
$$\begin{array}{ll} E{\left\|{\hat{\mu }}^{OLS}-{\hat{\mu }}^{UR}\right\|}^{2} & \geq E\left({\left\|{\hat{\mu }}^{OLS}-{\hat{\mu }}^{UR}\right\|}^{2}1_{H}\right)\\ \; & \geq E\left(n{\hat{\lambda }}_{0}\sum_{j:{\hat{\beta }}_{j}^{OLS}\beta_{j}^{uni}<0}{\left({\hat{\beta }}_{j}^{OLS}\right)}^{2}1_{H}\right)\\ \; & \geq n\lambda_{0}^{\prime }\sum_{j:\beta_{j}=0,\beta_{j}^{\mathrm{uni}}\neq 0}E\left({\left({\hat{\beta }}_{j}^{\text{OLS}}\right)}^{2}1_{\left\{{\hat{\beta }}_{j}^{\text{OLS}}\beta_{j}^{\mathrm{uni}}<0\right\}}1_{H}\right). \end{array}$$

Take any j such that $\beta_{j}=0$ and $\beta_{j}^{\mathrm{uni}}\neq 0$. Note that

(C.13)
$$E\left({\left({\hat{\beta }}_{j}^{\text{OLS}\;}\right)}^{2}1_{\left\{{\hat{\beta }}_{j}^{\text{OLS}}\beta_{j}^{\mathrm{uni}}<0\right\}}1_{H}\right)=E\left({\left({\hat{\beta }}_{j}^{\text{OLS}}\right)}^{2}1_{\left\{{\hat{\beta }}_{j}^{\text{OLS}}\beta_{j}^{\mathrm{uni}}<0\right\}}1_{F}\right)\;\;-E\left({\left({\hat{\beta }}_{j}^{\text{OLS}}\right)}^{2}1_{\left\{{\hat{\beta }}_{j}^{\text{OLS}}\beta_{j}^{\mathrm{uni}}<0\right\}}1_{F\cap E^{c}}\right)$$

By Lemma 5, the inequality (C.11), and the fact that the event F depends only on X, we get

(C.14)
$$\begin{array}{l} \text{E}\left({\left({\hat{\beta }}_{j}^{\text{OLS}}\right)}^{2}1_{\left\{{\hat{\beta }}_{j}^{\text{OLS}}\beta_{j}^{\text{uni}}<0\right\}}1_{F}\right)=\text{E}\left(\text{E}\left({\left({\hat{\beta }}_{j}^{\text{OLS}}\right)}^{2}1_{\left\{{\hat{\beta }}_{j}^{\text{OLS}}\beta_{j}^{\text{uni}}<0\right\}}\;|\;X\right)1_{F}\right)\\ \;\geq \frac{\sigma^{2}}{2n{\lambda_{1}}^{\prime }}\mathbb{P}\left(F\right)\geq \frac{\sigma^{2}}{2n{\lambda_{1}}^{\prime }}\left(1-C_{3}e^{-C_{4}n}\right). \end{array}$$

On the other hand, by the Cauchy-Schwarz inequality, (C.10), and Lemma 2,

(C.15)
$$\begin{array}{ll} E\left({\left({\hat{\beta }}_{j}^{OLS}\right)}^{2}1_{\left\{{\hat{\beta }}_{j}^{OLS}\beta_{j}^{uni}<0\right\}}1_{F\cap E^{c}}\right) & \leq {\left[E\left({\left({\hat{\beta }}_{j}^{OLS}\right)}^{4}1_{F}\right)\;P\left(E^{c}\right)\right]}^{1/2}\\ \; & \leq {\left[E\left(E\left({\left({\hat{\beta }}_{j}^{OLS}\right)}^{4}|X\right)1_{F}\right)\right]}^{1/2}\sqrt{p}\;e^{-\frac{1}{2}C_{0}n}\\ \; & \leq {\left(\frac{3\sigma^{4}}{n\lambda_{0}^{\prime }}\right)}^{1/2}\sqrt{p}\;e^{-\frac{1}{2}C_{0}n}\\ \; & =\frac{\sqrt{3p}\;\sigma^{2}}{\sqrt{{\lambda_{0}}^{\prime }}}e^{-\frac{1}{2}C_{0}n}. \end{array}$$

Combining equations (C.12), (C.13), (C.14), and (C.15), we get

$$\begin{array}{ll} E{\left\|{\hat{\mu }}^{OLS}-{\hat{\mu }}^{UR}\right\|}^{2} & \geq n{\lambda_{0}}^{\prime }q\left(\frac{\sigma^{2}}{2n{\lambda_{1}}^{\prime }}\left(1-C_{3}e^{-C_{4}n}\right)-\frac{\sqrt{3r}\sigma^{2}}{\sqrt{{\lambda_{0}}^{\prime }}}e^{-\frac{1}{2}C_{0}n}\right)\\ \; & =\frac{\sigma^{2}q{\lambda_{0}}^{\prime }}{2{\lambda_{1}}^{\prime }}-C_{3}\frac{\sigma^{2}q{\lambda_{0}}^{\prime }}{2{\lambda_{1}}^{\prime }}e^{-C_{4}n}-\sqrt{3r{\lambda_{0}}^{\prime }}q\sigma^{2}ne^{-\frac{1}{2}C_{0}n}. \end{array}$$

By Lemma 3, this completes the proof.

**Table:**

(n.d.-a).	(n.d.-j).
(n.d.-b).	(n.d.-k).
(n.d.-c).	(n.d.-l).
(n.d.-d).	(n.d.-m).
(n.d.-e).	(n.d.-n).
(n.d.-f).	(n.d.-o).
(n.d.-g).	(n.d.-p).
(n.d.-h).	(n.d.-q).
(n.d.-i).	
